# YAP regulates cell mechanics by controlling focal adhesion assembly

**DOI:** 10.1038/ncomms15321

**Published:** 2017-05-15

**Authors:** Giorgia Nardone, Jorge Oliver-De La Cruz, Jan Vrbsky, Cecilia Martini, Jan Pribyl, Petr Skládal, Martin Pešl, Guido Caluori, Stefania Pagliari, Fabiana Martino, Zuzana Maceckova, Marian Hajduch, Andres Sanz-Garcia, Nicola Maria Pugno, Gorazd Bernard Stokin, Giancarlo Forte

**Affiliations:** 1 International Clinical Research Center (ICRC), St Anne's University Hospital, CZ-65691 Brno, Czech Republic; 2 CEITEC MU, Masaryk University, CZ-65691 Brno, Czech Republic; 3 Faculty of Medicine, Department of Biology, Masaryk University, CZ-62500 Brno, Czech Republic; 4 Faculty of Medicine and Dentistry, Institute of Molecular and Translational Medicine, Palacky University Olomouc, Hněvotínská 1333/5, CZ-77515 Olomouc, Czech Republic; 5 Faculty of Pharmacy, Division of Pharmaceutical Biosciences, University of Helsinki, Viikinkaari 5 E, FI-00014 Helsinki, Finland; 6 Institute of Advanced Biomedical Engineering and Science, Tokyo Women's Medical University, 8-1 Kawada-Cho, Shinjuku-Ku, Tokyo JP-162-8666, Japan; 7 Laboratory of Bio-inspired and Graphene Nanomechanics, Department of Civil, Environmental and Mechanical Engineering, University of Trento, I-38123 Trento, Italy; 8 Ket-Lab, Italian Space Agency, Via del Politecnico snc, 00133 Rome, Italy; 9 School of Engineering and Materials Science, Queen Mary University of London, Mile End Road, UK-E1 4NS London, UK; 10 Department of Biomaterials Science, Institute of Dentistry, University of Turku, FI-20014 Turku, Finland

## Abstract

Hippo effectors YAP/TAZ act as on–off mechanosensing switches by sensing modifications in extracellular matrix (ECM) composition and mechanics. The regulation of their activity has been described by a hierarchical model in which elements of Hippo pathway are under the control of focal adhesions (FAs). Here we unveil the molecular mechanism by which cell spreading and RhoA GTPase activity control FA formation through YAP to stabilize the anchorage of the actin cytoskeleton to the cell membrane. This mechanism requires YAP co-transcriptional function and involves the activation of genes encoding for integrins and FA docking proteins. Tuning YAP transcriptional activity leads to the modification of cell mechanics, force development and adhesion strength, and determines cell shape, migration and differentiation. These results provide new insights into the mechanism of YAP mechanosensing activity and qualify this Hippo effector as the key determinant of cell mechanics in response to ECM cues.

## Abstract (web summary)

The transcriptional co-activator YAP is known to operate downstream of mechanical signals arising from the cell niche. Here the authors demonstrate that YAP controls cell mechanics, force development and adhesion strength by promoting the transcription of genes related to focal adhesions.

Cells are in constant isometric tension with the extracellular matrix (ECM), an equilibrium of forces needed to ensure to adopt the shape and volume suited to exert their function^1^,^2^. On a larger scale, this condition keeps organ functionality, while changes in the mechanical balance between the cells and the surrounding *milieu* result in tissue malfunctioning or malignant transformation^3^,^4^.

The ability of cells to perceive ECM mechanics and spread is associated to Hippo pathway effectors Yes-associated protein (YAP) and WW domain-containing transcription regulator protein 1 (WWTR1 or TAZ) shuttling to the nucleus to exert their co-transcriptional activity^5^,^6^. By binding to cell- and context-specific transcription factors, YAP/TAZ contribute to ECM remodelling^7^,^8^,^9^.

Focal adhesions (FAs), the main hub for cell mechanosensing, act as a bridge between integrin-ECM connection and the cytoskeleton^10^. Changes in the signals propagated through FAs have been reported in malignant cells and are essential for tumour cell spreading^11^.

YAP/TAZ nuclear activity is correlated to the stability of actin cytoskeleton and cell tension, as controlled by myosin light chain II and Rho GTPase pathways^12^,^13^,^14^. Integrin-FA signalling has been recently suggested to control Hippo pathway by phosphorylating large tumour suppressor (LATS) kinases through Src^15^. These results predicted a hierarchical mechanism by which Hippo effectors behave as downstream sensors of ECM mechanics through integrin-FA signalling and by perceiving cytoskeleton stability. Here we describe the molecular basis of the crosstalk among the different cell mechanosensing systems and propose a model by which YAP directly regulates FA assembly and cell mechanics.

## Results

### Cell area controls YAP shuttling regardless of FA assembly

**Figure 1:**
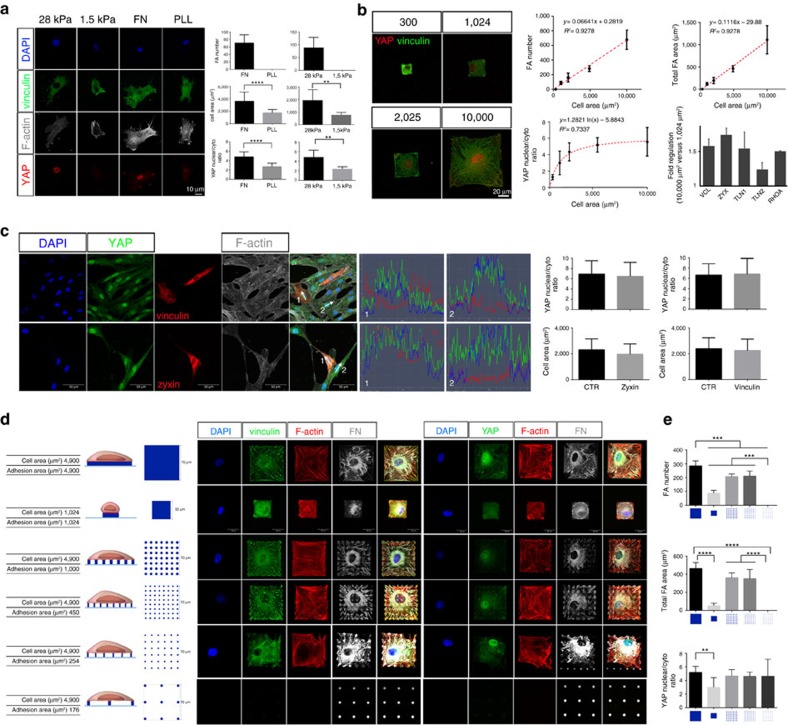
YAP nuclear shuttling is determined by cell area. (**a**) Confocal images of AD-MSCs cultured onto elastically supported surfaces (ESS) with 28, 1.5 kPa or fibronectin- and poly-L-lysine-coated glass slides. Cells were stained with the indicated antibodies: anti-vinculin (green), anti-YAP (red). F-actin was decorated with Alexa Fluor 647 Phalloidin (white) and nuclei were counterstained with 4′,6-diamidino-2-phenylindole (DAPI). Graphs: quantification of the number of FAs per cell (FA number), cell area and YAP nuclear/cytoplasmic ratio in AD-MSCs cultured on ESS, fibronectin or poly-L-lysine (*n*=10, 3 technical replicates, Mann–Whitney test, ****P<0.0001 and **P<0,001). (**b**) Confocal analysis of single AD-MSCs cultured onto micropatterned glass slides with 300, 1,024, 2,025 and 10,000 μm^2^ areas and coated with fibronectin. Cells were stained with anti-YAP (red), and anti-vinculin (green). Graphs: quantification of FA number, total FA area and YAP nuclear/cytoplasmic ratio in single AD-MSCs grown onto fibronectin-coated surfaces with increasing adhesion areas. Bottom right: Quantitative RT–PCR analysis of vinculin (*VCL*), zyxin (*ZYX*), talin1 (*TLN1*), talin2 (*TLN2*), *RHOA* genes in AD-MSCs cultured onto 10,000 versus 1,024 μm^2^. The results are expressed as mean fold regulation obtained in 2 independent experiments and the bar indicates the s.d. (**c**) Confocal images and analysis of AD-MSCs transfected with vinculin-venus or RFP-zyxin and stained for YAP. F-actin was decorated with Alexa Fluor 647 Phalloidin (white). Graphs: quantification of YAP nucleus/cytoplasm ratio and cell area in transfected cells. (**d**) Left: Annotation of micropattern properties (cell area, adhesion area), schematic side view of single cell onto fibronectin-coated micropatterned glass slides, schematic top view of fibronectin distribution in the micropatterns. Fibronectin-covered area is indicated in blue. Center: confocal images of single AD-MSCs grown onto micropatterns stained with anti-vinculin (green). Right: confocal images of single AD-MSCs grown onto micropatterns stained with anti-YAP (green). In both panels cells are stained with anti-fibronectin (white), Alexa Fluor 546 Phalloidin and DAPI. (**e**) Graphs: quantification of FA number, total FA area and YAP nuclear/cytoplasmic ratio in single cells cultured onto fibronectin-coated surfaces. All error bars are s.d. (*n*=10, 3 technical replicates, ****P<0.0001, *** *P*<0.001 and **P<0.01, as calculated by Kruskal–Wallis test followed by post hoc Dunn's test for multiple comparison).

Considering recent evidence suggests possible interplay between Hippo pathway and FAs^15^,^16^,^17^, we investigated the correlation between YAP nuclear localization and the presence of FAs. To this end, we cultured adipose tissue-derived mesenchymal stem cells (AD-MSCs) onto fibronectin (FN)-coated elastically supported surfaces of different stiffness (28 and 1.5 kPa) or onto glass surfaces coated either with FN or poly-L-lysine (PLL). FN coating onto the stiff surface (28 kPa) promotes FA assembly, whereas the exposure of cells to PLL abrogates FA formation regardless of substrate stiffness. In addition, FN is not able to foster FA arrangement on soft (1.5 kPa) surfaces. Interestingly, in all the conditions in which FA formation was prevented (PLL or soft surfaces), YAP was excluded from the nucleus and cell area was significantly reduced as compared with the controls, consistent with the absence of FAs in the cell^18^ (*n*=10, 3 technical replicates, Mann–Whitney test, ****P<0.0001 and **P<0,001; Fig. 1a). Remarkably, when single cell area was controlled by growing AD-MSCs onto FN-coated micropatterns forcing the cell to acquire given sizes (300, 1,024, 2,025 and 10,000 μm^2^), YAP nuclear expression correlated with the presence of vinculin spikes. In fact, both YAP nucleus/cytoplasm ratio and the number of FAs increased steadily with the size of the cell. Moreover, the expression of FA genes vinculin (*VCL*), zyxin (*ZYX*), talin 1 (*TLN1*) and talin 2 (*TLN2*) was found increased in cells having larger (10,000 μm^2^) as compared with smaller surface areas (1,024 μm^2^). Spread cells also displayed enhanced *RHOA* expression as compared with the confined controls. These results suggested that the expression of FA-related genes and the formation of the multiprotein complexes were correlated to YAP nuclear localization and controlled by cell spreading (Fig. 1b).

Given the correlation between FA presence and nuclear YAP, we asked whether YAP nuclear accumulation was determined by cell size or by the formation of FAs. Therefore, we overexpressed two main FA components—vinculin and zyxin—in AD-MSCs and detected no significant change in nuclear YAP and cell surface area (Fig. 1c). Moreover, we designed single cell tools based on surface micropatterning by which cell area could be tailored independently of the availability of FN spots. By this means, cells were induced to maintain the same surface area while contacting a different number of adhesion sites. This experimental setting allowed us to finely tune FA number regardless of cell size. Within the same experiments, we were also able to compare cells having different cell area but constant adhesion area, as mediated by FN distribution (Fig. 1d). As expected, when the adhesion area was reduced (4,900 versus 1,024 μm^2^), the number of FAs dramatically dropped (284±38 versus 88±21, mean±s.d.) and the ratio between nuclear and cytoplasmic YAP decreased accordingly (5.19±0.88 versus 2.99±1.44). On the contrary, when cell area was kept constant (4,900 μm^2^) but the adhesion area decreased (1,000 and 450 μm^2^), no significant effect on YAP nuclear localization was detected (5.20±0.88 versus 4.66±0.94 or 4.32±1.08, respectively), independently of the reduction in FA number (from 283.9±38.14 to 207.0±20.30 and 210.7±36.80, respectively; Fig. 1e). When the adhesion area was reduced to 254 μm^2^ in cells preserving constant area (4,900 μm^2^), no FA spike organization was detected, while YAP nuclear localization was not significantly affected (*n*=10, 3 technical replicates, Dunn's test, ****P<0.0001, ***P<0.001 and **P<0.01). Lower adhesion areas (176 μm^2^) did not allow cell adhesion. These results pointed at YAP nuclear accumulation as a function of cell area rather than FA formation, as controlled by ECM adhesion. Moreover, they raised the possibility that the acquisition of cell area could directly affect YAP localization and function, independently of FA assembly.

### YAP tunes FAs and cell stiffness in response to cell area

To explore the connection among cell area, YAP and FAs, we knocked down YAP in AD-MSCs by short hairpin RNA (shYAP) and controlled cell area through FN-coated micropatterns. The same experiment was performed with cells in which YAP paralogue protein TAZ (shTAZ) was silenced, as cells grown onto PLL (lacking FAs) displayed a significant reduction in TAZ nuclear localization as compared with those cultured on FN (Supplementary Fig. 1). The selective silencing of *YAP* or *TAZ* was confirmed in quantitative PCR analysis (Supplementary Fig. 2a).

**Figure 2:**
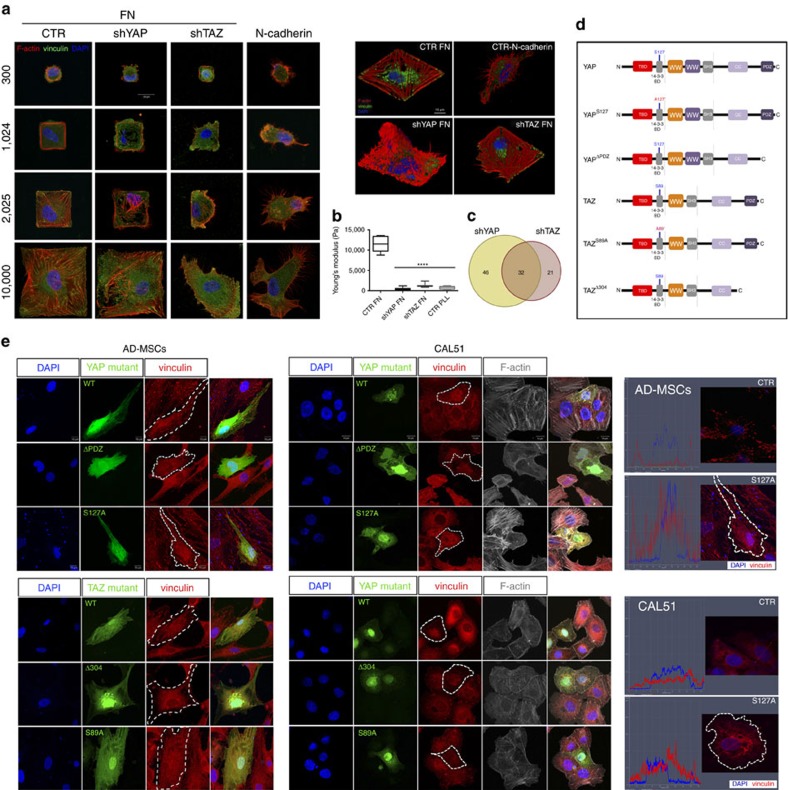
YAP regulates FA formation and cell stiffness in response to cell area in AD-MSCs. (**a**) Left: confocal analysis of individual cells grown onto 300, 1,024, 2,025, 10,000 μm^2^ micropatterns coated with either fibronectin or N-cadherin. Right: three-dimensional (3D) reconstruction of individual cells grown onto 10,000 μm^2^. Cells were stained with: anti-vinculin (green) Alexa Fluor 546 Phalloidin and 4′,6-diamidino-2-phenylindole (DAPI). Cells were infected with viral particles encoding for either control short hairpin RNA (CTR), short-hairpin RNA targeting YAP or TAZ (shYAP, shTAZ, respectively). (**b**) Graph: Young's modulus analysis of single AD-MSCs, shYAP and shTAZ onto micropatterns coated with FN or PLL. Values are shown as median±min/max (*n*=24, 3 technical replicates, Kruskal–Wallis test followed by Dunn's test, ****P<0.0001). (**c**) Venn diagram showing the overlap between genes significantly regulated in shYAP and shTAZ cells as obtained by quantitative PCR arrays (see Supplementary Fig. 3a). (**d**) Schematic structure of YAP (S127A, ΔPDZ) and TAZ (S89A, Δ304) mutants used for the transfections. (**e**) Confocal images of focal adhesion distribution, as stained by anti-vinculin (red) and DAPI in AD-MSCs (left) and CAL51 (centre) transfected with the indicated YAP and TAZ mutants. Right: Representative image analysis of vinculin expression in AD-MSCs and CAL51 transfected or not with S127A YAP mutant. Transfected AD-MSCs were stained with anti-vinculin antibody (red). Transfected CAL51 cells were stained with anti-vinculin (red), Alexa Fluor 647 Phalloidin (white) and DAPI. Transfected cells are identified by GFP expression and highlighted by dashed lines.

Although control cells (CTR FN) were able to acquire the given size and shape over FN micropatterns, shYAP cells displayed impaired FA maturation and acquired a bulging phenotype with deranged F-actin cytoskeleton protruding in spikes. On the contrary, TAZ-deprived cells preserved a correct cytoskeleton arrangement and developed FAs, while losing their ability to spread on the given surface: (Fig. 2a, Supplementary Fig. 2b and Supplementary Movies 1–3). Given these results, we decided to impair both YAP and TAZ nuclear localization in the cells, by culturing them onto N-cadherin micropatterns. The use of this experimental condition was suggested by the evidence that the concomitant silencing of both YAP and TAZ did not allow the cells to adhere.

Consistent with the model of cadherin–catenin system sequestering both YAP and TAZ at the cell–cell interaction site^19^,^20^, AD-MSCs grown onto N-cadherin-coated surface and stained with an antibody recognizing both YAP and TAZ displayed no nuclear localization of the proteins. In such conditions, FA formation was completely abrogated, cytoskeleton was disrupted and cells failed to acquire the given shape and size (Fig. 2a and Supplementary Fig. 2c). FAs and cytoskeleton are required to control cell mechanics in response to ECM cues^10^,^21^. Therefore, we decided to measure the stiffness of shYAP and shTAZ AD-MSCs by atomic force microscopy (AFM). The analysis of single cell Young's Modulus clarified that both shYAP and shTAZ cells were significantly less stiff than the control and comparable to cells lacking FAs (Fig. 2b). Elasticity value for control cells grown onto FN-coated glass surfaces was 11,518±1,786.5 Pa, whereas values calculated for shYAP, shTAZ on FN, and control cells cultured onto PLL-coated glass were 371±200.35, 1,205±70.5 and 755.7±358.45 Pa, respectively (*n*=24, 3 technical replicates, Dunn's test, ****P<0.0001). Owing to cell detaching from the surface, it was not possible to measure neither cells in which both YAP and TAZ were depleted nor cells grown onto N-cadherin micropatterns.

As YAP- and TAZ-silenced cells displayed different phenotypes, we analysed the transcriptional activity of the two proteins as well as their impact on cell differentiation potential. The paralogue proteins exhibited different transcriptional profiles, like shown by PCR array analysis (Fig. 2c and Supplementary Fig. 3a,b, the full gene list is provided in Supplementary Data 1) and distinct adipogenic differentiation potential as compared to the control cells and among themselves (Supplementary Fig. 3b). Altogether, the results indicated that—besides acting as mechanosensors by perceiving ECM mechanics, similar to that previously shown^7^—YAP and TAZ are responsible of regulating cell stiffness in AD-MSCs. Moreover, the data suggested that YAP activity was exerted by controlling the assembly of FAs.

YAP/TAZ are known to exert their function as transcriptional co-activators mainly by binding TEAD transcription factor^22^. Therefore, we transiently overexpressed in two different cell types (AD-MSCs and Cal51) constructs coding for mutants of YAP and TAZ having a nuclear (YAP^S127A^, TAZ^S89A^) or cytoplasmic (YAP^ΔPDZ^, TAZ^Δ304^) localization^23^,^24^,^25^. YAP^S127A^ and TAZ^S89A^ mutants cannot be phosphorylated by LATS1/2 kinases and thus are predominantly located in cell nucleus and transcriptionally active^26^. On the other hand, YAP^ΔPDZ^ and TAZ^Δ304^ mutants lack the PDZ domain, which is required for nuclear shuttling^22^ and thus are sequestered to the cytoplasm (Fig. 2d). Staining transfected cells for FA protein vinculin highlighted an increase in the signal and an altered distribution of FA spikes induced by the transcriptionally active YAP mutant (YAP^S127A^). On the contrary, no change in FA expression was triggered by TAZ constitutively active mutant (TAZ^S89A^) and the cytoplasmic mutants of both proteins (YAP^ΔPDZ^ and TAZ^Δ304^) (Fig. 2e).

Overall, these results suggested that the nuclear presence and the co-transcriptional activity of YAP were requested to promote FA formation.

### YAP directly regulates the expression of FA-related genes

**Figure 3:**
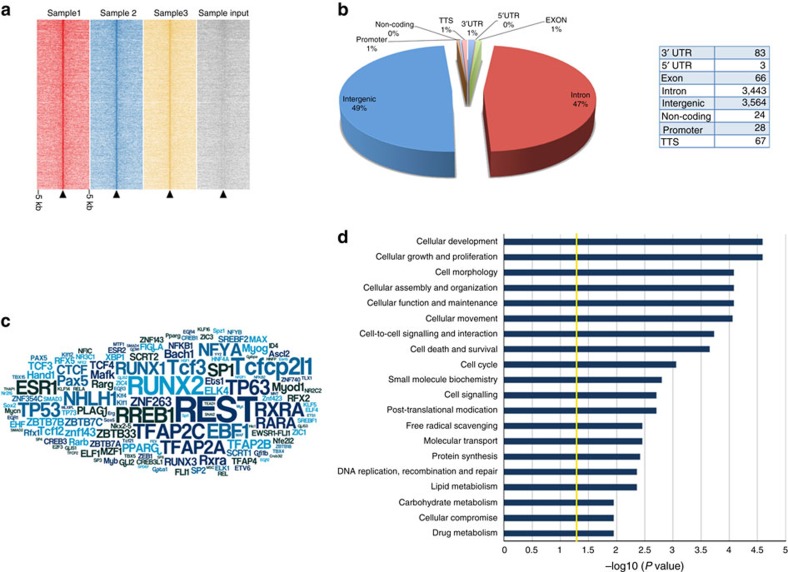
YAP directly targets FA genes in CAL51 cells. (**a**) Heatmap representing the consistent enrichment of YAP binding sites in three independent preparations (A1, A2, A3) of CAL51 cells. (**b**) Graph: location annotation of YAP DNA binding sites as obtained by ChIP-Seek web tool. Table: location annotation of YAP DNA binding sites reported as absolute values. (**c**) word cloud representation of the most significantly represented transcription factors known to bind the sequences identified as YAP targets. Font size is inversely correlated to −log10(*P* value). (**d**) Table: Gene functional annotation of the 7,278 hits identified as YAP targets on immunoprecipitated chromatin with their respective *P*-value.

To understand whether YAP nuclear activity determines a direct activation of FA genes, we performed chromatin immunoprecipitation followed by deep sequencing (ChIP-Seq) analysis of YAP-specific DNA binding sites in CAL51 cells. A total of 7,278 peaks accounting for 4,321 unique hits were identified, this number being consistent with previous analysis performed on different cell types^9^,^27^. A very limited amount of YAP-binding sites were promoters, most of them being intergenic or intronic (Fig. 3a,b). The list of all targets identified is reported in Supplementary Data 2, whereas the methodology adopted and quality metrics parameters are reported in Supplementary Fig. 4. As YAP does not possess any ability to bind DNA *per se*, we analysed the transcription factor binding motifs known to target the sites identified by ChIP-Seq. The analysis yielded a number of potential partners involved in YAP co-transcriptional activity (Supplementary Data 3), with some of them being well-known to interact with YAP (TEAD1, p73, RUNX1, RUNX2 and PPARG), while others being newly related to YAP1, such as REST, RREB1 and ESR1 (Fig. 3c). Molecular function annotation of the identified hits clarified most of the genes targeted by YAP are involved in cell development, cell growth and proliferation and in the acquisition of cell morphology, roles which have been previously associated to YAP activity (Fig. 3d)^27^. More interestingly, among the functions revealed by the analysis, those related to the formation and number of FAs displayed high scores (*P*=1.85 × 10^−4^ and *P*=1.88 × 10^−3^, respectively, *n*=3, Fisher's exact test), while also categories correlated to cytoskeleton organization and formation were represented (*P*=6.15 × 10^−4^) (Supplementary Data 4). The ability of YAP to target FA genes was corroborated by comparing our original data with the results published by an independent research group in MDA-MB-231 breast cancer line^27^ (*P*=1.3 × 10^−4^; Easy score; Supplementary Fig. 5a).

### YAP controls FA integrity downstream of RhoA GTPase pathway

**Figure 4:**
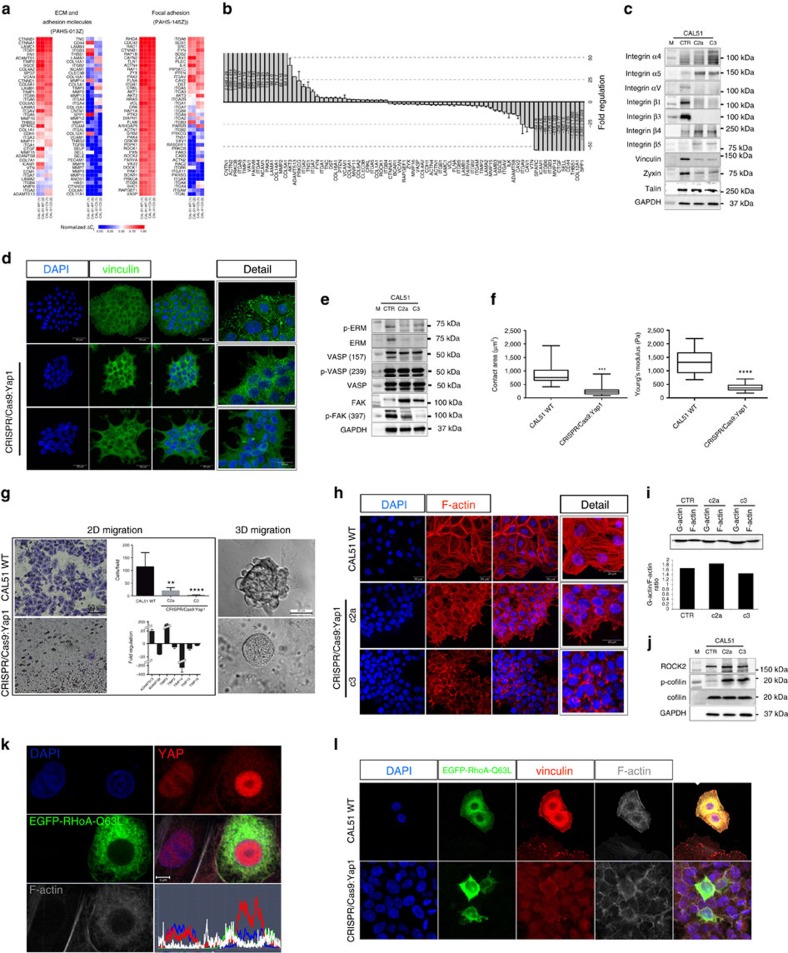
YAP regulates focal adhesion/cytoskeleton integrity downstream of RhoA. (**a**) Heatmap representing the RNA expression levels by normalized Δ*C*_t_ values obtained in two qRT–PCR independent replicates of YAP mutant clone C3 as compared with CAL51 WT. (**b**) Graph: barplot representing mean value±s.d. of up- and downregulated genes with a fold change higher than 2.0 in YAP mutant clone C3 as compared to CAL51 WT. (**c**) Western blot analysis of the indicated focal adhesion (FA) proteins; *n*=3. (**d**) Representative confocal images of WT CAL51 and YAP mutant clones C2a and C3, showing vinculin staining (green). (**e**) Protein analysis of the indicated cytoskeleton- and FA-associated proteins in WT CAL51 and YAP mutant clones; *n*=3. (**f**) Graphs: quantification of cell contact area and Young's Modulus in WT CAL51 and YAP-defective clone C3. Values are shown as median±min/max (*n*=24, 3 technical replicates, Welch's *t*-test, ***P<0.001 and **** *P*<0.0001,). (**g**) Box: Representative brightfield image of WT CAL51 and C3 clone migrated through ECM-coated transwell membrane (8 μm) and stained with crystal violet after 24 h, quantification is shown in the upper graph. The data represent the mean value±s.d. (12 random fields, 2 technical replicates, **P<0.01 and ***P<0.001, Kruskal–Wallis test followed by *post hoc* Dunn‘s test). Bottom: qRT–PCR array analysis of genes involved in ECM remodelling in YAP mutant cells (C3) as compared with the control. The threshold was set at 2.0. Right: Representative brightfield image of CAL51 WT and YAP mutant cells C3 grown in three-dimensional (3D) Matrigel for 120 h. (**h**) Representative confocal images showing the arrangement of F-actin in WT Cal51 and mutant clones C2a and C3 stained with Alexa Fluor 546 Phalloidin. (**i**) Representative western blot quantification of G-actin/F-actin ratio in WT CAL51 and YAP mutant clones. (**j**) Representative western blot analysis of the indicated proteins; *n*=3. (**k**) Representative confocal image of WT CAL51 cell transfected with EGFP-RhoA-Q63L and stained with anti-YAP antibody (red), Alexa Fluor 647 Phalloidin (white). Image analysis shows the nuclear/cytoplasmic distribution of YAP protein in non-transfected and transfected cells. (**l**) Confocal images of WT CAL51 and mutant clone C3 transfected with EGFP-RhoA-Q63L and stained with anti-vinculin antibody (red), Alexa Fluor 647 Phalloidin (white).

Given the evidence that YAP can directly target FA-related genes, we generated CAL51 stable mutant cell lines lacking YAP by CRISPR/Cas9 technology. The characterization of the cell lines is shown in Supplementary Fig. 5b–e. As predicted by ChIP-Seq analysis, YAP mutant clones underwent a switch in the expression of genes involved in FA formation and cell–ECM interaction (Fig. 4a). Remarkably, the expression of known targets of YAP like *CTGF* was consistently reduced in mutant cells. More importantly, the expression profile of integrin subunits was found altered in YAP-depleted cells. *ITGA1*, *ITGA4* and *ITGAV*, which were also identified as direct targets of YAP in ChIP-Seq analysis, were consistently altered in YAP mutant cells (Fig. 4b). In addition, changes in *ITGA5*, *ITGB3* and *ITGB1* were confirmed at the protein level (Fig. 4c) and suggested a higher degree of complexity other than the direct gene targeting by YAP. These changes were accompanied by a clear reduction in key FA structural proteins vinculin and zyxin, and the displacement of talin (Fig. 4d,e and Supplementary Fig. 6a). Similar results were obtained in HEK293 YAP-depleted cells (Supplementary Fig. 6b).

As a result of the inability of developing FA spikes, cells lacking YAP encountered a dramatic shift in cell morphology with colonies failing to spread and acquire their regular shape (Supplementary Fig. 6c). Cell shape is determined by the tight interplay between FAs and cytoskeleton, with docking proteins Ezrin/Radixin/Moesin (ERM) and Enabled/vasodilator-stimulated phosphoprotein (VASP) being responsible for the stabilization of the interphase^21^,^28^,^29^,^30^,^31^. Mutant cells displayed a significant reduction in ERM expression and its phosphorylated form together with decreased phosphorylation on S157 site of VASP, responsible for its localization to the FAs. No change in VASP phosphorylation at S239 site was detected, a modification needed for cytoskeleton stabilization^29^. As expected for cells lacking FA spikes, a marked reduction in FAK autophosphorylation on Tyr397 site was found in YAP mutant cells, indicating a lack of integrin heterodimerization, a step needed for FA assembly^32^ (Fig. 4e).

The integrity of the interphase between FAs and cytoskeleton is required for the cell to establish a functional connection with the ECM during morphogenesis and migration^33^. As expected, mutant cells lost the ability to spread over the ECM, as measured by cell contact area (*A*_WT_=873.1±331.0 μm^2^ versus *A*_CRIPR/CAS9:YAP1_: 254.4±153.8 μm^2^), became softer (*E*_WT_=1320.62±409.78 Pa versus *A*_CRIPR/CAS9:YAP1_: 405.28±143.29 Pa) (*n*=24, 3 technical replicates, *P<0.001 and **P<0.0001, Welch's *t*-test) and failed to migrate in two-dimensional and three-dimensional conditions (two technical replicates, Dunn‘s test, ****P<0.001 and **P<0.01) (Fig. 4f,g). This effect was accompanied in YAP-deficient cells by the modulation of genes encoding for proteins involved in the degradation of ECM (Fig. 4g and Supplementary Movie 4).

Rho/ROCK axis is considered to be the main mechanosensing pathway perceiving ECM mechanics^34^,^35^, being able to control not only FA formation^36^ but also actin polymerization dynamics by keeping cofilin severing protein in the phosphorylated, inactive form^30^. As Rho/ROCK have been found to control YAP shuttling in different reports^37^ and no impairment in *RhoA*, *ROCK1* and *ROCK2* gene expression was induced by YAP-depleted cells, we next asked whether these effects are connected.

YAP-deficient cells displayed a rearrangement in F-actin cytoskeleton in a similar manner to what we described in shYAP AD-MSCs (Fig. 4h), but no evident changes in the tubulin network (Supplementary Fig. 6d). Western blot analysis of the ratio between F- and G-actin clarified no impairment in actin bundle polymerization occurred in cells lacking YAP (Fig. 4i). The cells had unaltered ROCK2 levels and accumulated the phosphorylated inactive form of actin severing protein cofilin (Fig. 4j). These results clarified that the lack of FA structural and docking proteins prevented the anchorage of cell cytoskeleton to the membrane, and that Rho/ROCK role in actin dynamics was independent of YAP activity.

On the other hand, we asked whether YAP nuclear shuttling was required for Rho/ROCK acknowledged control over FA formation. The treatment with pharmacological inhibitors interfering with Rho/ROCK pathway at different levels, (Y27362, LIMKi3 and cytochalasin D) caused YAP shuttling to the cytoplasm, triggered cytoskeleton disassembly and the disappearance of FAs (Supplementary Fig. 7a,b). On the contrary, the overexpression of RhoA-Q63L constitutively active mutant increased YAP nuclear localization (Fig. 4k) and resulted in a marked increase in FA signal together with the formation of stress fibres. Rho hyperactive mutant failed to induce the formation of FAs in YAP-deprived lines (Fig. 4l). These data clarified that YAP was necessary downstream of Rho/ROCK to govern FA formation and cell–matrix interaction.

### Cell mechanics is determined by YAP-induced FA assembly

**Figure 5:**
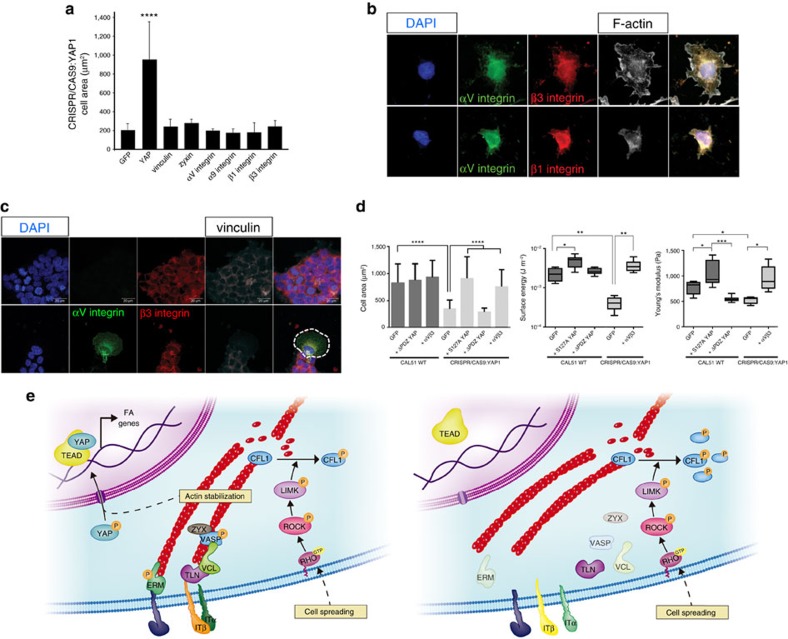
YAP controls cell biophysical properties and the interaction with ECM through αVβ3 integrin. (**a**) Graph: YAP mutant cell contact area. YAP-depleted cells were transfected as to express the indicated proteins and the contact area of transfected cells was evaluated and compared to the GFP-transfected cells. (*n*=26, 3 technical replicates, ****P<0.0001, as calculated by Kruskal–Wallis test followed by post hoc Dunn's test). Barplot represents mean value±sd. (**b**) Confocal representative images of YAP-deprived cells transfected with either αVβ1 or αVβ3 integrins. Cell F-actin was stained with Alexa Fluor 647 Phalloidin (white) and nuclei counterstained with 4′,6-diamidino-2-phenylindole (DAPI) (blue). (**c**) Confocal images of CAL51 mutant cells transfected with either αV (green), β3 (red) or co-transfected with αVβ3 integrins (co-stained by red and green). Double transfected cell is highlighted by a dashed line. Cells were decorated with vinculin (white) and nuclei counterstained with DAPI (blue). (**d**) Graphs: comparison of the indicated biophysical properties of WT CAL51 (grey) and YAP mutant clone C3 (pale grey), transfected or not to overexpress the reported proteins. Cell area: *n*=10, 3 technical replicates, ****P<0.0001, as calculated by Kruskal-Wallis test followed by post hoc Dunn's test. The barplot represents mean value±sd. The surface energy is given in logarithmic scale. Young's Modulus, surface energy: *n*=10, 3 technical replicates, *P<0.05, **P<0.01 and ***P<0.001 as calculated by Welch's ANOVA test followed by post hoc Games-Howell test. Values are shown as median±min/max. (**e**) Model proposing YAP control over focal adhesion (FA) assembly. Left: Cell area controls RhoA activity to phosphorylate cofilin (CFL1) and maintain the integrity of actin fiber cytoskeleton, which triggers YAP nuclear shuttling. In the nucleus, YAP promotes the transcription of genes encoding for proteins participating in FA assembly. Right: when YAP is lost, Integrin subunit remodelling occurs, docking proteins like vinculin, zyxin, ERM and VASP are removed from the interphase between FAs and F-actin, and the anchorage of cytoskeleton to the membrane is disrupted. All error bars are s.d.

Given the plethora of DNA targets hit by YAP and connected to FA assembly, we asked what are the key genes targeted by YAP that trigger the formation of FAs, thus determining cell spreading and mechanics. Therefore, we complemented YAP mutant cells with single components of the FA complex with a structural function that were depleted or mismatched in mutant cells (vinculin, zyxin, talin, αV-, β1- and β3-integrins). As a control for proteins that were not affected, we also included α9 integrin. As expected, although YAP transduction in mutant cells restored some degree of vinculin and zyxin expression, single FA proteins such as vinculin, zyxin, talin, α9-, αV-, β1- and β3-integrin failed to induce any change in cell ability to interact with the ECM, as measured by cell surface area and cell morphology (*n*=26, 3 technical replicates, Dunn's test, ****P<0.0001). (Fig. 5a and Supplementary Fig. 8). In previous reports, αVβ1 and αVβ3 integrins—two major subsets of integrins displaced in YAP-depleted cells—were associated to cell mechanics, adhesion and migration^38^,^39^. Therefore, we co-transfected mutant cells with αVβ1 and αVβ3-integrins. No significant increase in mutant cell surface area was induced by αVβ1, whereas a significant and consistent restoration was obtained with αVβ3 transfection (*A*_αVβ3_=751.5.1±316.8 μm^2^ versus *A*_CRIPR/CAS9:YAP1_: 346.6±161.1 μm^2^), similar to the values obtained by re-expressing S127A YAP (A_S127A_=903.8±404.8 μm^2^) constitutively active mutant and wild type cells (*A*_WT_=823.1±355.2 μm^2^; Fig. 5b,c) (*n*=3 independent experiments, Dunn's test, ****P<0.0001). As a result, YAP-defective cells transfected with αVβ3 integrins developed a higher surface energy (*γ*_αVβ3_ : (3.7±1.3) × 10^−3^ μm^− 2^ versus *γ*_CRIPR/CAS9:YAP1_: (4.2±1.6)−10^−4^ J μm^−2^, *n*=3 independent experiments, Games–Howell test *P<0.05 and **P<0.01) and became significantly stiffer than the mutant cells (*γ*_αVβ3_: 952.8±248.5 Pa versus *γ*_CRIPR/CAS9:YAP1_: 533.9±80.9 Pa, *n*=3 independent experiments, Games–Howell test *P<0.05 and ***P<0.001) when measured in AFM.

## Discussion

The search for the processes by which YAP/TAZ exert their acknowledged mechanosensing activity recently led to a model in which two different upstream pathways partially overlap with Hippo pathway to control their nuclear shuttling and thus regulate their role as transcriptional co-activators. Small GTPase Rho indirectly controls YAP/TAZ nuclear localization by promoting the formation of actin bundles and stress fibres in response to the spreading of the cell over ECM^12^,^40^. Moreover, YAP/TAZ upstream kinase LATS is targeted by integrin-FAK signalling through Src^15^. These results depicted a role for Hippo effectors as a hub for different signalling pathways sensing ECM composition and mechanics through cytoskeleton stability. Here we demonstrate that: (1) YAP nuclear localization is controlled by cell area through Rho/ROCK activation and independent of FA formation; and (2) YAP transcriptionally controls FA formation and cytoskeleton stability, in turn determining cell mechanics and the degree of cell adhesion to the ECM.

Through a single-cell tool based on micropatterns able to control FA formation independently of cell size^41^, we clarified that YAP nuclear presence in AD-MSCs does not depend on the formation of FAs, but it is rather controlled by the area of the cell itself. Cell spreading over ECM directly controls *RHOA* gene expression and activation and this event is crucial to stabilize cell cytoskeleton by inhibiting the F-actin severing protein cofilin through phosphorylation by LIMK^35^. As confirmed in previous studies^12^, YAP acts downstream of Rho/ROCK and is sensitive to F-actin bundle polymerization. Conversely, the integrity of cytoskeleton actin bundles was not affected *per se* by YAP depletion, as shown by the unaltered ratio between F- and G-actin, and the sustained phosphorylation of cofilin severing protein, thus indicating that Rho/ROCK role in actin dynamics was independent of YAP activity.

The activation of Rho GTPase pathway is also required to foster the maturation of FAs in cells in contact with the ECM^36^. The anchorage of cell cytoskeleton to the FAs is of paramount importance to control cell shape and mechanics, with docking proteins such as vinculin, talin, zyxin, ERM and VASP being primarily involved in coupling cytoskeleton to integrins^21^,^28^,^29^,^30^,^31^. The lack of YAP in the cell caused the complete absence of the typical FA spikes highlighting the inability of YAP mutant cells to interact with the surrounding ECM. As a control, cells overexpressing the constitutively active and nuclear form of YAP (S127A YAP) showed an increased FA formation. YAP-depleted cells showed a reduced expression of FA stabilizing proteins ERM, vinculin and zyxin, and showed altered localization of actin docking protein VASP. The decreased expression and phosphorylation of ERM, a set of proteins providing structural linkage between transmembrane components and actin filaments^42^ together with the decreased phosphorylation on S157 site of VASP—a protein entitled to stabilize the interaction among FA docking proteins and actin cytoskeleton^29^—accounted for the failure in promoting the anchorage between the cytoskeleton and FAs.

Our ChIP-Seq analysis clarifies that the stability of FA-cytoskeleton interphase is directly under the control of YAP: YAP co-transcriptional function, as determined by cell area through Rho/ROCK-mediated cytoskeleton assembly, directly targets DNA binding sites responsible for the production of a number of proteins involved in FA and cytoskeleton assembly and crucial to cell-ECM interaction. These results, obtained in breast cancer CAL51 cells, are confirmed in other cell types by an unbiased meta-analysis performed on ChIP-Seq data previously obtained by other research groups^9^,^27^.

YAP displayed a specific DNA-binding signature for genes encoding for proteins involved in cytoskeleton stabilization (*CAPZA1*, *CAPZA2*, *ABRA*, *ACTRT1*, *CKAP4*, *CKAP4* and *TLN2*) and connection to the membrane (*PCDH15*, *CTNNA2*, *CTNNA3*, *CTNNB1*, *CTNND2*, *FAT3*, *PCDH7*, *PCDH17*, *PCDH10*, *PCDH20*, *CDH8*, *CDH5*, *CDH2*, *CDH20* and *CDH26*). Among YAP-targeted genes, those related to dystrophin/sarcoglycan complex (*SGCD*, *SGCG*, *ZSC1*, *SNTB1* and *IMPG2*) have also been identified in the fetal heart^13^ as YAP targets and directly related to the stabilization of the cytoskeleton.

Interestingly, YAP depletion resulted in the rearrangement of integrin expression pattern. Integrin expression profile modulates cell migration in response to ECM cues, with αVβ1 being responsible for random cell migration, whereas αVβ3 induce persistent directional cell migration^43^. The latter has also been shown to increase cell stiffness and is currently being targeted by etaracizumab monoclonal antibody in clinical studies aimed at hindering cancer propagation^44^. Although not being detected by our analysis as a direct target of YAP, β3 integrin is among the genes displaced by YAP knockout and, when re-expressed in combination with its partner αV, restored cell mechanical properties and the ability to spread. This evidence reinforces the indication of YAP as a target for cancer molecular therapy^45^.

The evidence that YAP-depleted cells encountered a significant reduction in their stiffness qualified YAP as a pivotal component of the mechanosensing apparatus and depicted a model in which YAP co-transcriptional activity is required for the synthesis of docking and stabilizing proteins allowing cell tension generation. Not surprisingly, the absence of adhesion complexes in YAP mutant cells resulted in their impaired ability to get a grip on the surrounding ECM. Indeed, cells lacking YAP displayed lower adhesion energy and reduced contact area when interacting with elements of the ECM. As a proof-of-concept, mutant cells failed to migrate and invade the surrounding matrix, when challenged in two-dimensional and three-dimensional assays.

YAP has been historically described to exert overlapping/redundant functions with its paralog protein TAZ^37^. Although TAZ depletion performed similarly in terms of cell mechanics in our cell models, it did not affect FA dynamics, this being an exclusive feature of YAP. Moreover, the mild observed upregulation of TAZ in YAP-silenced cells was incapable to counteract FA loss^46^. The apparent discrepancy between YAP and TAZ activities could be explained by the slight but consistent differences in the transcriptional and functional signatures of YAP and TAZ reported here and by others^46^,^27^. Solving this puzzle deserves further investigations.

Altogether, the data provided emphasize the role of YAP in controlling cell mechanics and describe a model in which cell spreading over the surrounding ECM modulates YAP transcriptional activity through Rho/ROCK axis to determine FA-cytoskeleton remodelling (Fig. 5e). When taken together with recent reports describing YAP as a regulator of ECM mechanics^8^, our findings point at YAP as the master regulator of cell–ECM interaction.

## Methods

### Cell culture and differentiation

ASC52telo, hTERT immortalized AD-MSC cells (ATCC SCRC-4000) were purchased from American Type Culture Collection (ATCC, Manassas, USA). HEK293 cells were kindly provided by Dr V. Pekarik (Department of Physiology, Masaryk University, Brno, Czech Republic). CAL51 cells were a gift of Dr L. Krejčí (Department of Biology, Masaryk University). Cells were cultured in DMEM medium 4.5 g l^−1^ Glucose (DMEM high Glucose, Lonza, Basel, Switzerland) supplemented with 10% fetal bovine serum, 2 mM L-glutamine and 100 U ml^−1^ penicillin/streptomycin. YAP and TAZ knockdown was performed in AD-MSCs by using a lentiviral vector short hairpin RNA, (shYAP, shTAZ; Santa Cruz Biotechnology, Dallas, USA), according to the manufacturer's instructions. Control cells were transfected with Control short hairpin RNA Lentiviral Particles (Santa Cruz Biotechnology). Briefly, 50% confluent AD-MSCs were cultured on 24-well plates and incubated with retroviral particles (multiplicity of infection: 1) in the presence of 5 μg ml^−1^ polybrene (Santa Cruz Biotechnology). One day after infection, the medium was replaced with fresh medium and cells were allowed to recover for 48 h. Infected cells were selected by adding 4 μg ml^−1^ puromycin (Santa Cruz Biotechnology) to the culture medium until the experiments were performed. AD-MSC adipogenic differentiation was assessed by culturing cells in complete differentiation medium (Thermo Fisher Scientific, Waltham, USA), according to the manufacturer's instructions. Three days after induction, lipid droplets were stained using AdipoRed, counterstained with 4′,6-diamidino-2-phenylindole (Thermo Fisher Scientific) and visualized by confocal microscopy. Nine independent fields were acquired at low magnification and the ratio of cells containing lipid droplets was calculated.

For the inhibition of Rho/ROCK pathway, cell were treated with Y27632 (10 μM, Sigma-Aldrich, St. Louis, USA), LIMKi3 (20 μM, Tocris Bioscience, Bristol, UK), cytochalasin D (1 μM, Sigma-Aldrich), or the corresponding amount of DMSO diluted in complete media for 24 h and then fixed and stained as described afterwards.

### Cell transfection

Cell transfection and co-transfection were performed by using Lipofectamine 3000 (Thermo Fisher Scientific). The plasmids pEGFP-C3-hYAP1 (17843, gift from Marius Sudol), 2 × FLAGhYAP1 (17791, gift from Marius Sudol), 2 × FLAGhYAP1-S127A (17790, gift from Marius Sudol), pLX304-YAP1_PDZ (59147, gift from William Hahn), 3 × Flag pCMV5-TOPO TAZ wild type (WT) (24809, gift from Jeff Wrana), 3 × Flag pCMV5-TOPO TAZ (S89A) (24815, gift from Jeff Wrana), 3 × Flag pCMV5-TOPO TAZ (∂304) (24810, gift from Jeff Wrana), pcDNA3-EGFP (13031, gift from Doug Golenbock), pcDNA3-EGFP-RhoA-Q63L (12968, gift from Gary Bokoch), EGFP-N3 Integrin alpha9 (Plasmid 13600, gift from Dean Sheppard), mEos2-Alpha-V-Integrin-25 (Plasmid 57345, gift from Michael Davidson and Catherine Galbraith), mCherry-Integrin-Beta1-N-18 (Plasmid 55064, gift from Michael Davidson), β3-integrin-YFP (Plasmid 26653, gift from Jonathan Jones) and 8 × GTIIC-lux (Plasmid 34615, gift from Stefano Piccolo) were obtained from Addgene. AD-MSCs and CAL51 were seeded into eight-well chamber slides. After 24 h, a preincubated mixture containing 20 μl of Opti-MEM, 0.2 ng of DNA, 0.6 μl of Lipofectamine 3000 and 0.4 μl P3000 reagent was added in each well. After 3 h, the medium was replaced and cells allowed to recover for 48 h before analyses.

For the AFM measurements, cells were transfected, sorted for the corresponding fluorescent channel and positive cells were seeded on FN-coated dishes and measured after 48 h similar to that described.

Luciferase assays analysis was quantified with Berthold CENTRO LB 960 Microplate Luminometer (Berthold Technologies GmbH, Austria).

### Generation of YAP mutant CAL51 lines

YAP-deficient Cal51 lines were produced by CRISPR/Cas9 technology. Guiding RNA was designed to hit exon 1 of *YAP1* gene, which is common to all nine YAP1 splicing variants (Supplementary Fig. 5b). Two sets of complementary single-stranded DNA oligonucleotides (YAP1_R1: 5′-CACCgtgcacgatctgatgcc-3′, YAP1_R2: 5′-AAACccgggcatcagatcgtgcac-3′, YAP1_F1: 5′-CACCGcatcagatcgtgcacgt-3′, YAP1_F2 5′-AAACcggacgtgcacgatctgatgC-3′) were cloned into pSpCas9(BB)-2A-GFP (PX458) (Addhene 48138, gift from Feng Zhang) and transfected into Cal51 cells using FuGENE HD (Promega Corporation, Wisconsin, USA) transfection reagent according to manufacturer's protocol. The next day, green fluorescent protein (GFP)-positive cells were FACS-sorted (MoFlo Astrios, Beckman Coulter, California, USA) as single cells into 96-well plate and clonally propagated. Genomic DNA was sequenced from both sides to map the deletion size (sequencing primers: 5′-gattggacccatcgtttgcg-3′, 5′-gtcaagggagttggagggaaa-3′, 5′-gaagaaggagtcgggcagctt-3′, 5′-gagtggacgactccagttcc-3′).

### Micropatterned substrates preparation

Fibronectin-coated or activated micropatterned slides with different area, shape or pattern (ref: 10–950–10–18; 10–950–00–18, or custom containing only 10,000 μm^2^ or 1,024 μm^2^ squares) were purchased from CYTOO (CYTOO, Grenoble, France). Laminin coating was performed by incubating activated slides with 20 μg ml^−1^ laminin (Sigma-Aldrich) at room temperature (RT) for 2 h. Cadherin coating was performed using a modification of the method described by Czöndör *et al*.^47^. Briefly, substrates were treated with 40 μg ml^−1^ poly-D-lysine (Merck Millipore, Billerica, USA) for 2 h, incubated with goat anti-human Fc antibody (Jackson Immunoresearch, West Grove, USA) in 0.2 M, pH 8.5 boric acid at RT for 5 h. Substrates were washed with borate buffer and incubated O/N at 4 °C with Recombinant Human E- or N-Cadherin Fc Chimeras (R&D Systems, Minneapolis, USA) in boric acid. Substrates were then washed and equilibrated in DMEM containing 10% fetal bovine serum for 1 h at 37 °C. AD-MSCs were seeded at 2 × 10^4^ cells per cm^2^ directly on the slides and cultured in standard culture conditions.

To investigate the effect of substrate stiffness on YAP and vinculin expression, cells were seeded onto Fibronectin-coated μ-Dish 35 mm, high elastically supported surface 28 and 1.5 kPa (iBIDI, Munich, Germany).

### Immunostaining and image analysis

Immunofluorescence staining was performed as previously described^48^. Briefly, cells were fixed in 4% paraformaldehyde in PBS for 15 min at RT and permeabilized with 0.1% Triton X-100 for 2 min. After incubation with primary antibodies, cells were incubated with the appropriate Alexa fluorochrome-conjugated secondary antibodies. F-actin was stained with Alexa Fluor 546 or 647-conjugated phalloidin. Nuclei were counterstained with 4′,6′-diamidino-2-phenylindole. Samples were embedded in ProLong Gold antifade reagent (Thermo Fisher Scientific) and visualized with Zeiss LSM 780 confocal microscope with × 40 (1.3 numerical aperture) and × 63 (1.4 numerical aperture) oil-immersion objective lenses. Z-stacks were acquired with the optimal interval suggested by the software, followed by the application of maximum intensity algorithm.

YAP nucleus/cytoplasm ratio was calculated using an *ad hoc* developed ImageJ plug-in with the following formula:


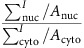


where 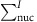 and 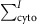 represent the sum of the intensity values for the pixels in the nuclear and cytoplasmic region respectively, and *A*_nuc_ and *A*_cyto_ the area of the corresponding regions.

Focal adhesion quantification was performed by ImageJ software as follows. All the images were acquired at the same magnification and resolution as described in (ref. 49). SUBTRACT BACKGROUND was applied to the channel corresponding to vinculin staining with a SLIDING PARABOLOID option and ROLLING BALL radius of 25 pixels. The images were enhanced by running CLAHE plug in (lock size=19, histogram bins=256, maximum slope=3, no mask and fast) followed by automatic BRIGHTNESS/CONTRAST and ENHANCE CONTRAST (saturated=0.35). Images were finally binarized using automatic THRESHOLD command (default settings) and particles analysed (size=0.30–15; circularity=0.00–0.99).

Primary antibodies used were as follows: rabbit anti-YAP (4912, Cell Signaling Technology, Danvers, USA), mouse anti-YAP/TAZ (sc-101199), rabbit anti-TAZ (sc-48805) (Santa Cruz Biotechnology), rabbit anti-Fibronectin (F3648), mouse anti-Talin (SAB4200041), mouse anti-Vinculin (V9131), mouse anti-Zyxin (Z0377), mouse anti-β-Tubulin (T5076) (Sigma-Aldrich) and mouse anti-Vinculin (ab18058, Abcam, Cambridge, UK). The full list of antibodies and dilutions can be found in Supplementary Data 5.

### Isolation of RNA and real-time quantitative PCR analysis

Total RNA was isolated using RNeasy Mini Kit (Qiagen, Hilden, Germany), according to the manufacturer's instructions. Complementary DNA was synthesized using the RT^2^ First Strand Kit (SABiosciences, Frederick, USA). The expression profile of genes involved in different pathways was analysed by the following RT^2^ Profiler PCR Arrays (Qiagen): MSCs (PAHS-082Z), Hippo signalling pathway (PAHS-172Z), human adherens junctions (PAHS-146Z), ECM and adhesion molecules (PAHS-013Z) and FAs (PAHS-145Z). RT–PCR was carried out on the LightCycler 480 Real-Time PCR System (Roche, Basel, Switzerland) using the following cycling parameters: 1 cycle at 95 °C for 10 min; 45 cycles at 95 °C for 15 s and 60 °C for 1 min. The internal panel of housekeeping genes set from the manufacturer was used for normalization of expression levels of individual genes and PCR-array data analysed by online resources from the manufacturer's website (http://www.sabiosciences.com/pcrarraydataanalysis. php) and statistical R-project software (http://www.r-project.org). Genes showing high coefficient of variation among replicas, as well as very low expression in all the experiments (35<*C*_t_<40) were discarded from the analysis. The results include the heatmaps of quantification cycles (*C*_t_) and the graphs of the mean and s.e.m. values of the fold regulation values obtained by analysing independently two samples per each experimental condition.

### Western blotting

Cells were lysed in RIPA buffer (Merck Millipore) with protease and phosphatase inhibitor cocktails (1%, both Sigma-Aldrich) on ice and then centrifuged at 13.000 g for 10 min at 4 °C. Supernatants were stored at −80 °C. Protein concentration was determined using the BCA method. Forty micrograms of proteins for each sample were loaded in on 10% polyacrylamide gels (Bio-Rad, Marnes-la-Coquette, France), pre-run at 40 V and then at 100 V. The fractionation into soluble, nuclear and cytoskeletal compartments was performed using a ProteoExtract Cytoskeleton Enrichment and Isolation Kit (Merck Millipore), according to the manufacturer's manual. G/F-actin ratio was quantified using the G-Actin/F-Actin *In Vivo* Assay Biochem Kit (Cytoskeleton, Denver, USA), according to the product specification. Proteins were transferred to a polyvinylidene difluoride membrane using the *Trans*-Blot Turbo transfer system (Bio-Rad). Membranes were blocked with 5% BSA in TBST, incubated with diluted primary antibody in 5% BSA in TBST at 4 °C O/N and then probed with the proper secondary HRP-linked antibody (Sigma-Aldrich) at RT for 1 h. ChemiDoc imaging system (Bio-Rad) was used to detect chemiluminescence. Band intensities were quantified using Bio-Rad Image Lab software.

Primary antibodies used were as follows: rabbit anti-YAP (4912), rabbit anti-YAP/TAZ (8418), rabbit anti-Integrin α4 (8440), rabbit anti-Integrin α5 (4705), rabbit anti-Integrin αV (4711), rabbit anti-Integrin β1 (9699), rabbit anti-Integrin β3 (13166), rabbit anti-Integrin β4 (4707), rabbit anti-Integrin β-5 (3629), rabbit anti-Cofilin (3313), rabbit anti-Phospho-Cofilin (Ser3) (5175), rabbit anti-Ezrin/Radixin/Moesin (3142), rabbit anti-Phospho-Ezrin (Thr567)/Radixin (Thr564)/Moesin (Thr558) (3726), rabbit anti-VASP (3132), rabbit anti-Phospho-VASP (Ser157) (3111), rabbit anti- Phospho-VASP (Ser239) (3114), rabbit anti-FAK (13009), rabbit anti-Phospho-FAK (Tyr397) (8556) (Cell Signaling Technology), rabbit anti-TAZ (PA1-46190), mouse anti-GAPDH-HRP conjugate (MA5-15738-HRP) (Thermo Fisher Scientific), mouse anti-Vinculin (ab18058), mouse anti-ROCK-2 (ab56661) (Abcam), mouse anti-Talin (SAB4200041), mouse anti-Zyxin (Z0377), mouse anti-β-Tubulin (T5076) (Sigma-Aldrich) and rabbit anti-Pan-Actin (AAN01, Cytoskeleton). The full list of antibodies and dilutions can be found in Supplementary Data 5. The original blots are shown in Supplementary Fig. 9.

### Scanning electron microscopy

Cal51 WT and YAP mutant cells were cultured in fibronectin-coated coverslips for two days, fixed with a solution of Glutaraldehyde 3% in 100 mM Cacodylate buffer and dehydrated in a series of increasing ethanol concentrations. Samples were mounted on aluminium stubs, sputter-coated with Palladium (JEOL JFC-1300, Tokyo, Japan) and imaged with a Benchtop Scanning Electron Microscope JEOL JCM-6000 (Tokyo, Japan).

### Chromatin immunoprecipitation

CAL51 cells were cultured in complete medium for 48 h. Chromatin was immunoprecipitated from three technical replicates using a ChIP-grade anti-YAP antibody (Cell Signaling Technologies) and following the manufacturer's instructions (Pierce Agarose ChIP Kit, Thermo Fisher Scientific). Control ChIP was performed by adding mouse immunoglobulins (IgG). The samples were eluted in 30 μl of eluting buffer and stored at -80 °C before the analysis.

### Library preparation and sequencing

Library preparation was performed and size distribution of each ChIP DNA sample was assessed by running a 1 μl aliquot on Agilent High Sensitivity DNA chip using an Agilent Technologies 2100 Bioanalyzer (Agilent Technologies). The concentration of each DNA sample was determined by using Quant-IT DNA Assay Kit- High Sensitivity and Qubit Fluorometer (Life Technologies). Ten nanograms of purified ChIP DNA were used as starting material for sequencing libraries preparation. Indexed libraries were prepared with TruSeq ChIP Sample Prep Kit (Illumina Inc.). Libraries were sequenced (single read, 1x50 cycles) at a concentration of 10 pM/lane on HiSeq 2500 (Illumina Inc.).

### ChIP-Seq data analysis

Data analysis was performed by Genomix4Life S.r.l (Baronissi, Italy). The raw sequence files generated (.fastq) underwent quality control analysis using FASTQC (http://www.bioinformatics.babraham.ac.uk). Reads were aligned to the human genome (assembly hg19) using bowtie^49^, allowing up to 1 mismatch and considering uniquely mappable reads. The reads of biological replicates and corresponding input samples were merged for peaks calling as previously described^50^. ChIP-Seq peaks were identified and analysed using HOMER^51^ (-F: 2.0, -L: 2.0 and -C: 1.0) with false discovery rate<0.01.

The assignment of YAP peaks to target genes was obtained by the web tool ChIPSeek^52^. Through this step, it was possible to assign peaks to transcription start site (by default defined from −1 kb to +100 bp), transcription termination site (by default defined from −100 bp to +1 kb), Exon (Coding), 5′-untranslated region (UTR) Exon, 3′ UTR Exon, Intronic or Intergenic. As some annotation overlap, the following order of priority was chosen for the assignment:

- Transcription start site (by default defined from −1 kb to +100 bp)
- Transcription termination site (by default defined from −100 bp to +1 kb)
- CDS exons
- 5′ UTR exons
- 3′ UTR exons
**CpG islands
**Repeats
- Introns
- Intergenic

Over-represented sequence motifs were defined according to motif descriptors of JASPAR database and computed using PScan-ChIP^53^. The following parameters were set:

- Organism: Homo Sapiens
- Assembly: hg19
- Background: Mixed
- Descriptors: Jaspar 2016

Nucleotide best occurrence was calculated by weblogo (http://weblogo.berkeley.edu/) by running Report Best Occurrences analysis on any given transcription factor.

Functional enrichment analysis was performed using IPA (IPA, QIAGEN, Redwood City, www.qiagen.com/ingenuity) by using the following parameters:

- Reference set: Ingenuity Knowledge Base (Genes Only)
- Relationship to include: Direct and Indirect
- Filter: Consider only molecules and/or relationship where (species=Human) AND (confidence=Experimentally Observed).

An unbiased comparison was performed between the single YAP targets obtained in the CAL51 ChIP-Seq analysis and the ones previously reported for MDA-MB-231 (ref. 27). The mutual targets enrichment for KEGG biological pathways was examined through the Database for Annotation, Visualization and Integrated Discovery 6.8. An *α*=0,01 for the EASE score *P*-value was used as a threshold.

### Single- and collective cell invasion assays

Single-cell invasion was assayed by culturing 30,000 CAL51 WT and YAP mutant cells on ECM pre-coated transwell chambers with 8 μm pores and allowed to migrate for 24 h, according to the manufacturer's instructions (ECM508 - Chemotaxis Cell Migration Assay, Merck Millipore). Cells in the bottom of the filters were stained with crystal violet, counted in twelve random fields and averaged for the replicate wells.

Growth factor-reduced Matrigel (BD Biosciences, New Jersey, USA) was diluted to 4 mg ml^**−1**^ in serum free-cold cell culture media. 10 μl Matrigel containing 1500 wild type or YAP-mutant CAL51 were transferred to each well of a μ-Slide (Ibidi, Martinsried, Germany). Matrigel was allowed to solidify at 37 °C for 20 min, before adding complete medium. Medium was changed after 3 days.

### Young's modulus mapping by AFM

Standard bioAFM microscope JPK NanoWizard 3 (JPK, Berlin, Germany) was used to perform force mapping procedure. The scanning-by-probe head (maximal visualization range 100-100-15 μm in *X*-*Y*-*Z* axis) of AFM microscope was placed on inverted optical microscope Olympus IX-81, × 10 objective was used to find proper area covered with cells and to place cantilever in the proper position for force mapping procedure. Plastic Petri dish (TPP, Trasadingen, Switzerland) with either the distilled water for instrument calibration or with cell culture was placed inside the Petri dish heater (JPK) pre-heated to 37 °C for 30 min. Non-coated silicon nitride AFM cantilever Hydra 2R-100N (AppNano, California, USA) equipped with pyramidal silicon tip was used for all the experiments. The probe was calibrated before each experiment as described below. Then the laser reflection sum was maximized, followed by centering of the laser detector. The AFM probe was introduced in contact with the surface during a standard process of landing. The sensitivity of the AFM setup was determined as a slope of the force-distance curve (FDC) measured by lifting the cantilever with Z-height of 450 nm, time per curve was 3 s. The sensitivity was found in the range 15.07-15.37 nm V^**−1**^, cantilever stiffness was calibrated by measurement of its thermal noise and resulted between 17.34 and 19.19 pN nm^**−1**^ for different days of experiments.

The bioAFM setting was identical for all the force mapping procedure. SetPoint value was 1.0 nN (relative to baseline value), time per curve 0.5 s, Z-length 15.0 μm, speed of curve recording 30 μm s^**−1**^, the FDCs were recorded with data sample rate of 2 kHz. The force mapping procedure was performed as step-by-step recording of FDCs in the network of 64 × 64 points on 100 × 100 μm covering area of single cell for AD-MSCs or colonies for CAL51. Force mapping process provides a network of FDC (dependency of tip-sample interaction force on tip height above the surface), so called force maps. The absolute value of Young's modulus can be determined by fitting the FDC by Sneddon equation^54^:


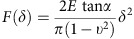


Where *F* is the measured force, *E* is Young's modulus, *ν* is Poisson ration (0.5 for incompressible materials), *δ* is tip-sample separation (obtained by correction of the cantilever height to its bending) and *α* is half-angle to face of pyramidal tip (reflects the tip geometry). Data processing module of the JPK software was used to process the maps of FDCs in a batch mode. Resulted Young's modulus maps were exported to be post-processed as described in the following chapter.

### AFM data analysis and cell mechanics evaluation

The elasticity maps obtained from measurements were pre-processed in open source SPM analysis software Gwyddion to extract geometrically coherent text tables of elasticity values and setpoint positions. Subsequently, data were processed by a Matlab (Mathworks, Natick, Massachussets, USA) script to remove outliers (for example, elasticity values coming from non-contacting points), select the portion of data inherent to the biological sample, observing the values distribution on histogram and calculate the statistical parameters of interest. Since cell-related elasticity value showed non normal distributions (that is, not passing a Lilliefors test), median and inter-quartile range were firstly computed as statistical parameters of confrontation. A finer extraction of the mean elastic modulus was performed with the Matlab distribution fitting tool, where data were found to best fit an inverse Gaussian distribution. Cell adhesion energy (*γ*) was calculated by using the JKR theory as described in ref. 55, assuming a conservative volume between the floating cell and the fully adherent one, and a zero load radius, through the following equation:


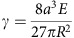


where *a* is the equivalent zero-loads contact radius (that is, the radius of a circle covering the same area) of an adherent cell, *R* is the measured contact of a floating cell and *E* is the elastic modulus measured as described above through AFM force mapping technique. Cell contact area was measured by ImageJ software region of interest tool on adherent CAL51 cells. The same software tools were used to extract the floating radius of the cell strains analysed. A sample number of 24, based on the sample number selected from the elasticity values, was considered selecting data with the minimum distance from their median.

### Statistical analysis

Data are presented as mean±s.d. and were calculated using the software package GraphPad Prism v. 6.0. For single cell analysis, a minimum of ten cells per sample was considered. The number of cells in 12 random fields per sample was evaluated in the migration assay. Statistical analyses were performed using Kruskal-Wallis test followed by post hoc multiple comparisons by Dunn's test. *P*>0.01 was not considered statistically significant. AFM data are shown in boxplot format, considering the median±min/max values calculated for each selected sample. For AD-MSCs data, which did not pass the Lilliesford normality test, statistical significance was calculated with the Kruskal-Wallis test followed by post hoc Dunn's test, and accepted for a *P*<0.05. For CAL51 data, passing the Lilliefors normality test but not homoscedasticity F-test, statistical significance was assessed by an unpaired two-tailed Welch's *t*-test, or Welch's analysis of variance test followed by Games–Howell test. Sample sizes were based on previously published experiments, in which statistical differences were identified.

### Data availability

ChIP-Seq analysis data were submitted to *ArrayExpress* database (https://www.ebi.ac.uk/arrayexpress/) where they can be accessed by the accession number: E-MTAB-5217. The data that support the findings of this study are available from the corresponding author upon reasonable request.

## Additional information

**How to cite this article:** Nardone, G. *et al*. YAP regulates cell mechanics by controlling focal adhesion assembly. *Nat. Commun.* **8,** 15321 doi: 10.1038/ncomms15321 (2017).

**Publisher's note**: Springer Nature remains neutral with regard to jurisdictional claims in published maps and institutional affiliations.

## Supplementary Material

Supplementary Information Supplementary Figures

Supplementary Movie 1 3D confocal reconstruction of single AD-MSCs grown onto 10,000 μm^2^ micropatterns and stained for vinculin (green), F-actin (red) and DAPI.

Supplementary Movie 2 3D confocal reconstruction of single shYAP AD-MSCs grown onto 10000 μm^2^ micropatterns and stained for vinculin (green), F-actin (red) and DAPI.

Supplementary Movie 3 3D confocal reconstruction of single shTAZ AD-MSCs grown onto 10000 μm^2^ micropatterns and stained for vinculin (green), F-actin (red) and DAPI.

Supplementary Movie 4 Representative 3D confocal reconstruction of spheroids obtained by culturing WT CAL51 and C3 cells (CRISPR/Cas9:YAP1) into growth factor reduced Matrigel^TM^ for 120 hours. Cells were stained for F-actin (red) and DAPI.

Supplementary Data 1 Full list of genes with the accession number analyzed by PCR arrays in AD-MSCs and CAL51 cells.

Supplementary Data 2 List of YAP targets as identified by ChIP-seq analysis in CAL51 WT cells.

Supplementary Data 3 List of transcription factors identified as potentially binding to the motifs obtained by ChIP-seq analysis in CAL51 WT cells.

Supplementary Data 4 Breakdown of the target genes as members of given functional clusters with their respective *P* value.

Supplementary Data 5 List of antibodies used in the study.
