# Supplementary material for: YAP regulates cell mechanics by controlling focal adhesion assembly
Source: Nat Commun. 2017 May 15;8:15321. doi: 10.1038/ncomms15321 (PMC5440673; doi:10.1038/ncomms15321)
Supplement: Supplementary Information — Supplementary Figures [file ncomms15321-s1.pdf]

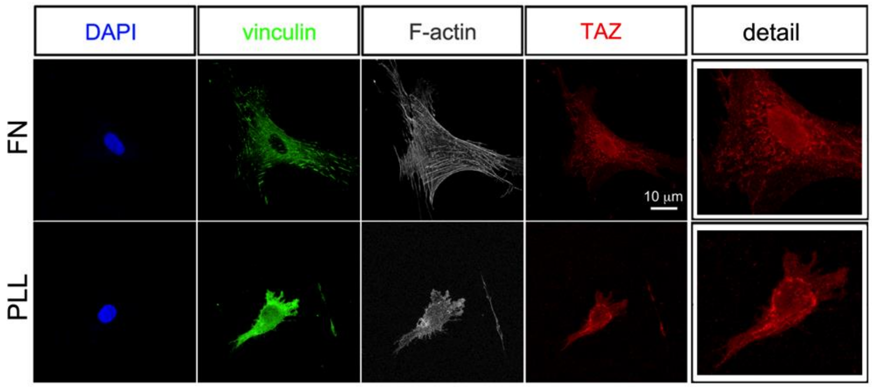

Supplementary Figure 1 │ Single AD-MSC cultured on fibronectin (FN) or poly-L-lysine (PLL)-coated glass slides and stained for vinculin (green), F-actin (white), TAZ (red). Nucleus is counterstained with DAPI.

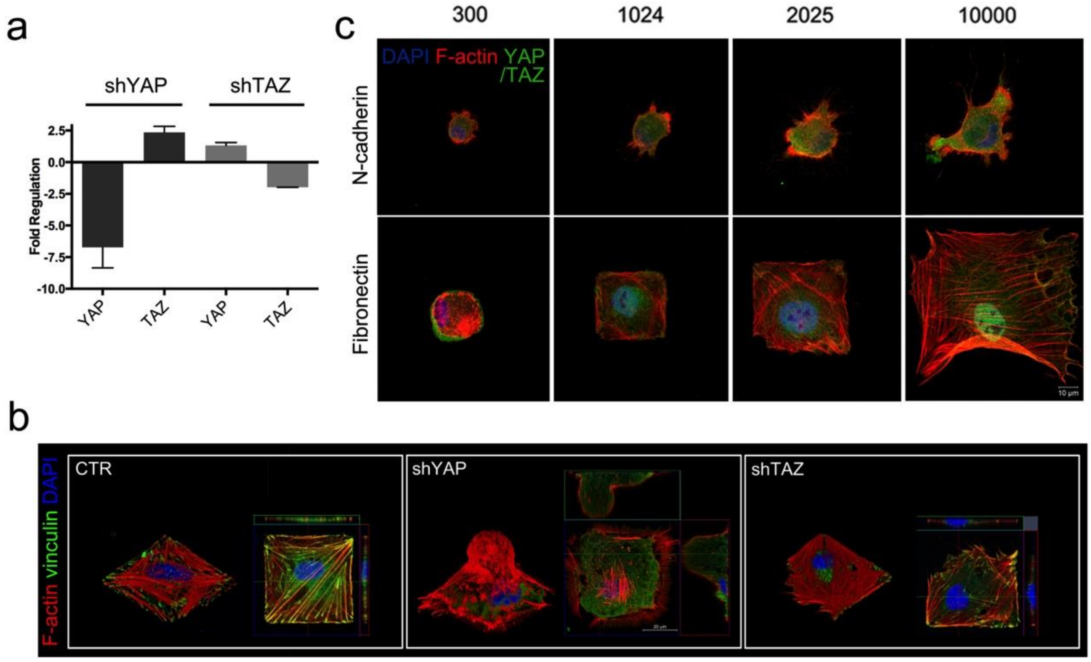

Supplementary Figure 2 │ **a,** Graph: Fold regulation of YAP and TAZ genes determined by qRT-PCR analysis in shYAP and shTAZ AD-MSCs. Results are expressed as fold regulation relative to the control. Data represent the mean value ± SD of 2 independent experiments. **b**, 3D confocal reconstruction and orthogonal projection of single AD-MSCs (CTR), shYAP, shTAZ grown onto 10,000 μm^2 micropatterns and stained with Alexa Fluor® 546 Phalloidin (red), anti-vinculin (green), and DAPI (blue). **c**, Representative confocal images of single AD-MSCs cultured on micropatterns coated with N-cadherin or fibronectin and having areas of 300, 1,024, 2,025, 10,000 μm^2. Cells were stained for YAP/TAZ (green), Alexa Fluor® 546 Phalloidin (red) and DAPI.

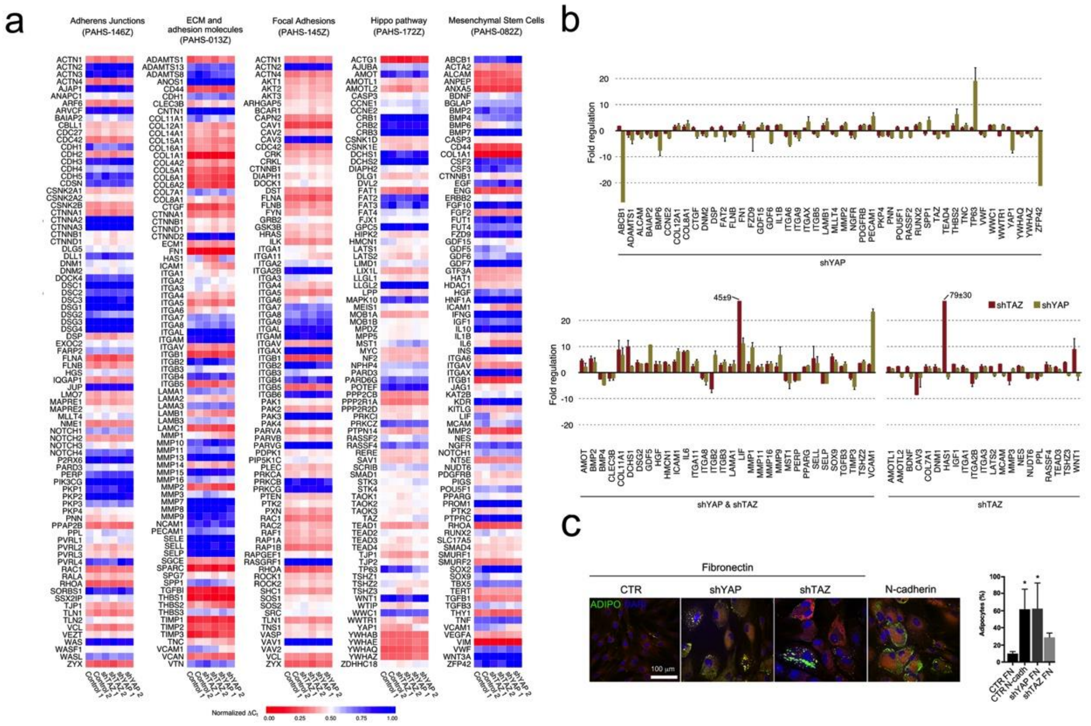

Supplementary Figure 3 │**a**, Gene expression data obtained by qRT-PCR array of genes belonging to Adherens Junctions (PAHS-146Z), ECM and adhesion molecules (PAHS-013Z), Focal adhesions (PAHS-145Z), Hippo Pathway (PAHS-172Z), and mesenchymal stem cells (PAHS-082Z) in shYAP and shTAZ AD-MSCs as compared to the control (CTR). The data represent the normalized ΔC_t of 2 independent experiments (indicated as 1 and 2). Detailed information of genes is shown in Supplementary Table 1. **b**, Graph: bar plot representation of up- and downregulated genes in shYAP, shTAZ AD-MSCs and common to both shYAP and shTAZ as compared to the control. The threshold was set to 2.0. Bars represent mean value ± SD. *c*, Representative confocal images of cells grown onto fibronectin (CTR, shYAP, shTAZ) or N-cadherin (CTR) exposed to adipogenic differentiation. Cells were fixed after 72 hours and stained with Adipored (ADIPO). The graph shows the quantification of lipid vacuoles-containing cells as quantified in 12 random fields. The data represent the mean value ± SD of 3 independent experiments. * *P*< 0,05.

a

Sample	# of Raw Reads	Q30	# Aligned reads (%)	# of duplicates reads (%)	rPhc
A1	55 395 061	37,67	39,703,217 (71.67)	3,937,197 (10)	3.32
A2	53 846 935	37,71	40,523,193 (72.26)	3,219,997 (8)	2.65
A3	56 151 169	37,66	38,229,318 (68.08)	3,380,273 (9)	2.83
Input_1	47 264 924	37,79	40,608,047 (85.92)	1,944,441 (5)	1.82
Input_2	64 881 642	37,88	56,675,909 (87.5)	3,119,445 (6)	1.76

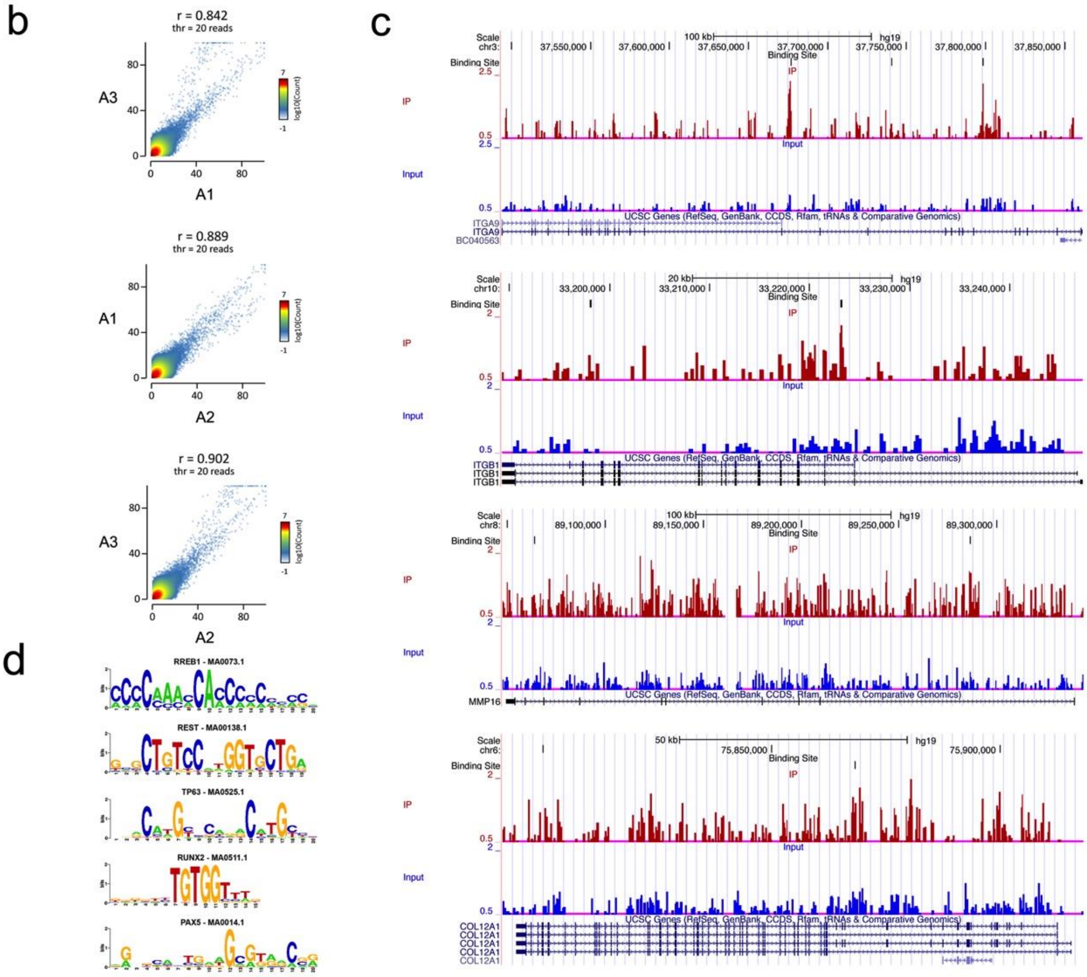

Supplementary Figure 4│a, Table summarizing the number of sequenced reads, Q30 value, number of aligned reads on reference genome, number of reads corresponding to PCR duplicates and rPhc value for IP and input samples. **b**, 2D histogram generated by Easeq (www.easeq.net) showing the number of genomic 100bp windows with the combination of the number of reads described on the axes. The correlated dataset were X-axes_name - Y-axes_name and a high degree of correlation results in a Pearson coefficient near 1, as indicated. Only window with more than 20 reads in one of the samples were used for calculating the Pearson coefficient. **c,** Genome Browser screenshots showing YAP occupancy tracks for ChIP-Seq experiment. Red track and blue track represent the distribution of YAP-IP and input samples on target genes, such as ITGA9, ITGB1, MMP16 and COL12A1 respectively. Merged data for IP and input samples are displayed. **d**, Motif analysis of top 5 over-represented transcription factors (RREB-1, REST, TP63, RUNX2, PAX5) predicted to interact with YAP.

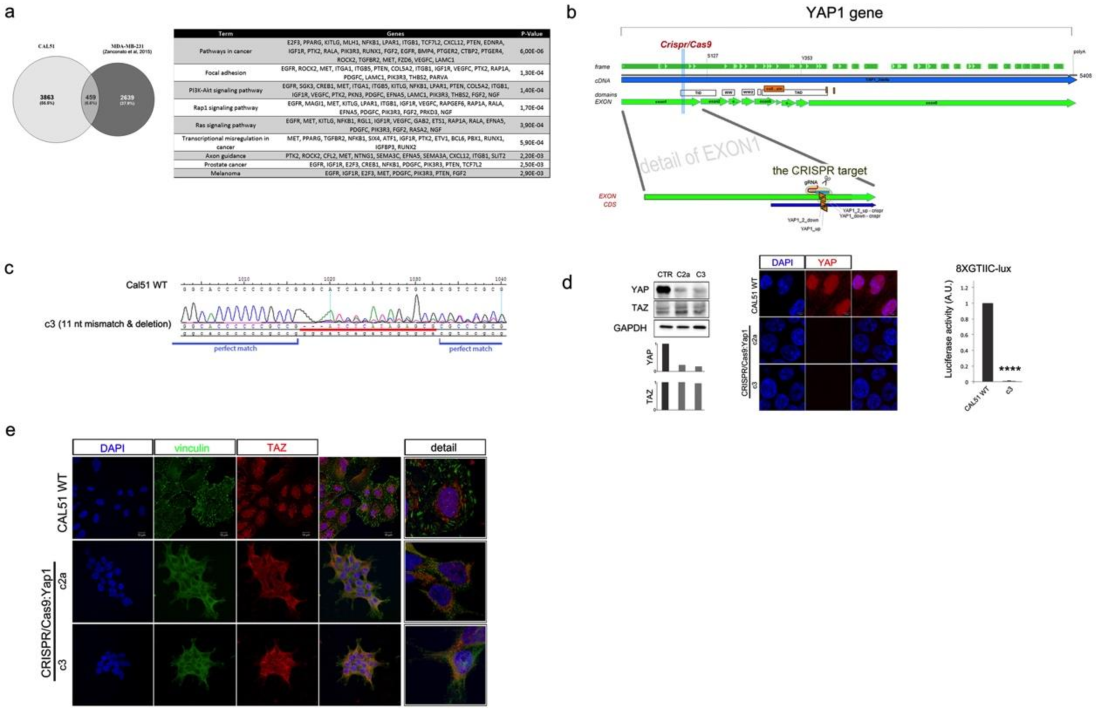

Term	Genes	P-Value
Pathways in cancer	E2F3, PPARG, KITLG, MLH1, NFKB1, LPAR1, ITGB1, TCF7L2, CXCL12, PTEN, EDNRA, IGF1R, PTK2, RALA, PIK3R3, RUNX1, FGF2, EGFR, BMP4, PTGER2, CTBP2, PTGER4, ROCK2, TGFBR2, MET, FZD6, VEGFC, LAMC1	6,00E-06
Focal adhesion	EGFR, ROCK2, MET, ITGA1, ITGB5, PTEN, COL5A2, ITGB1, IGF1R, VEGFC, PTK2, RAP1A, PDGFC, LAMC1, PIK3R3, THBS2, PARVA	1,30E-04
PI3K-Akt signaling pathway	EGFR, SGK3, CREB1, MET, ITGA1, ITGB5, KITLG, NFKB1, LPAR1, PTEN, COL5A2, ITGB1, IGF1R, VEGFC, PTK2, PKN3, PDGFC, EFNA5, LAMC1, PIK3R3, THBS2, FGF2, NGF	1,40E-04
Rap1 signaling pathway	EGFR, MAGI1, MET, KITLG, LPAR1, ITGB1, IGF1R, VEGFC, RAPGEF6, RAP1A, RALA, EFNA5, PDGFC, PIK3R3, FGF2, PRKD3, NGF	1,70E-04
Ras signaling pathway	EGFR, MET, KITLG, NFKB1, RGL1, IGF1R, VEGFC, GAB2, ETS1, RAP1A, RALA, EFNA5, PDGFC, PIK3R3, FGF2, RASA2, NGF	3,90E-04
Transcriptional misregulation in cancer	MET, PPARG, TGFBR2, NFKB1, SIX4, ATF1, IGF1R, PTK2, ETV1, BCL6, PBX1, RUNX1, IGFBP3, RUNX2	5,90E-04
Axon guidance	PTK2, ROCK2, CFL2, MET, NTNG1, SEMA3C, EFNA5, SEMA3A, CXCL12, ITGB1, SLIT2	2,20E-03
Prostate cancer	EGFR, IGF1R, E2F3, CREB1, NFKB1, PDGFC, PIK3R3, PTEN, TCF7L2	2,50E-03
Melanoma	EGFR, IGF1R, E2F3, MET, PDGFC, PIK3R3, PTEN, FGF2	2,90E-03

Supplementary Figure 5 | a, Meta-analysis of YAP target genes obtained in ChIP-seq for CAL51 compared to the ones previously reported for MDA-MB-231. The functional annotation for KEGG biological pathways of the common targets was carried out by using the Database for Annotation, Visualization and Integrated Discovery (DAVID 6.8). **b**, Schematic representation of the CRISPR/Cas9 strategy used to knock-out all splicing variants of YAP1 gene in CAL51 cells, by targeting the common exon 1. **c**, C3 mutant clone genomic DNA was sequenced from both sides to confirm the deletion in YAP exon 1. **d**, Left: representative Western blot analysis of YAP and TAZ expression in WT CAL51 and YAP mutant cells. Center: confocal analysis of YAP expression in wild type (WT) CAL51 cells and YAP mutant clones (C2a and C3). Cells were stained with anti-YAP antibody (red). Right: Analysis of YAP transcriptional activity. CAL51 WT and C3 clone cells were transfected with 8xGTIIC-lux reporter to analyze YAP-TEAD transcriptional activity. The data represent the mean value ± SD of 3 independent experiments. **** p<0,0001. **e**, TAZ expression in WT CAL51 and YAP-deficient clones C3 and C2a. Cells were stained with anti-vinculin (green), anti TAZ (red). Nuclei were counterstained with DAPI.

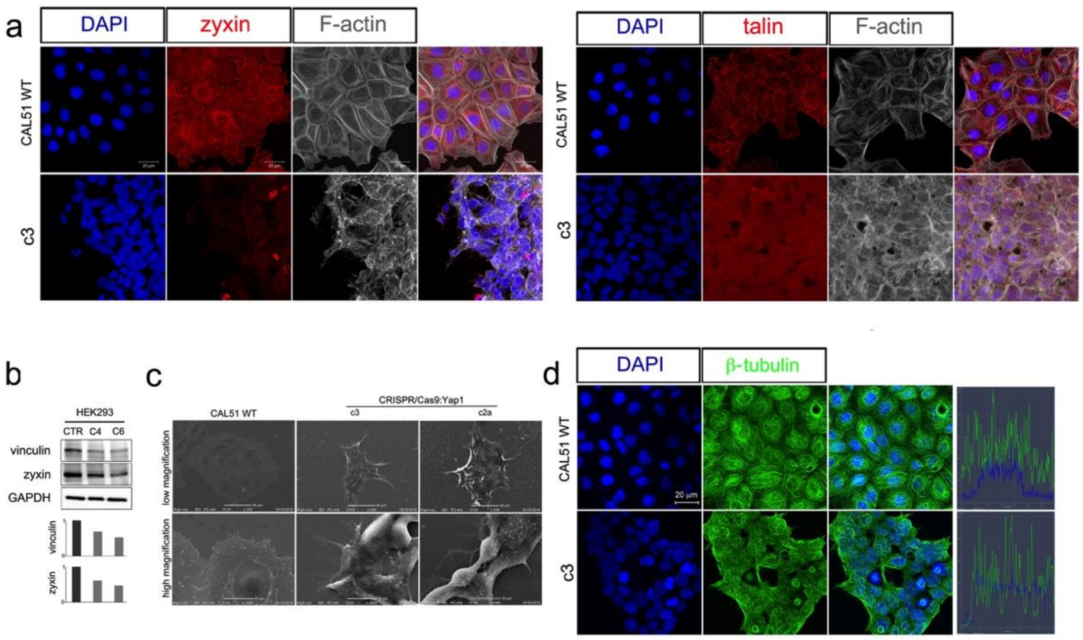

Supplementary Figure 6 │ a, Confocal images of CAL51 WT and YAP mutant clone C3 stained for either zyxin (red, left) or talin (red, right), Alexa Fluor® 647 Phalloidin (white) and DAPI. **b**, Representative Western blot analysis of the indicated proteins in HEK293 WT (CTR) and YAP mutant clones C4 and C6. **c**, Low (top) and high (bottom) magnification scanning electron microscopy (SEM) images of WT CAL51 and YAP mutants C2a and C3 showing colony morphology and adhesion processes. **d**, Confocal image analysis and quantification of β-tubulin expression in CAL51 and YAP-depleted clone C3. The cells are counterstained with DAPI.

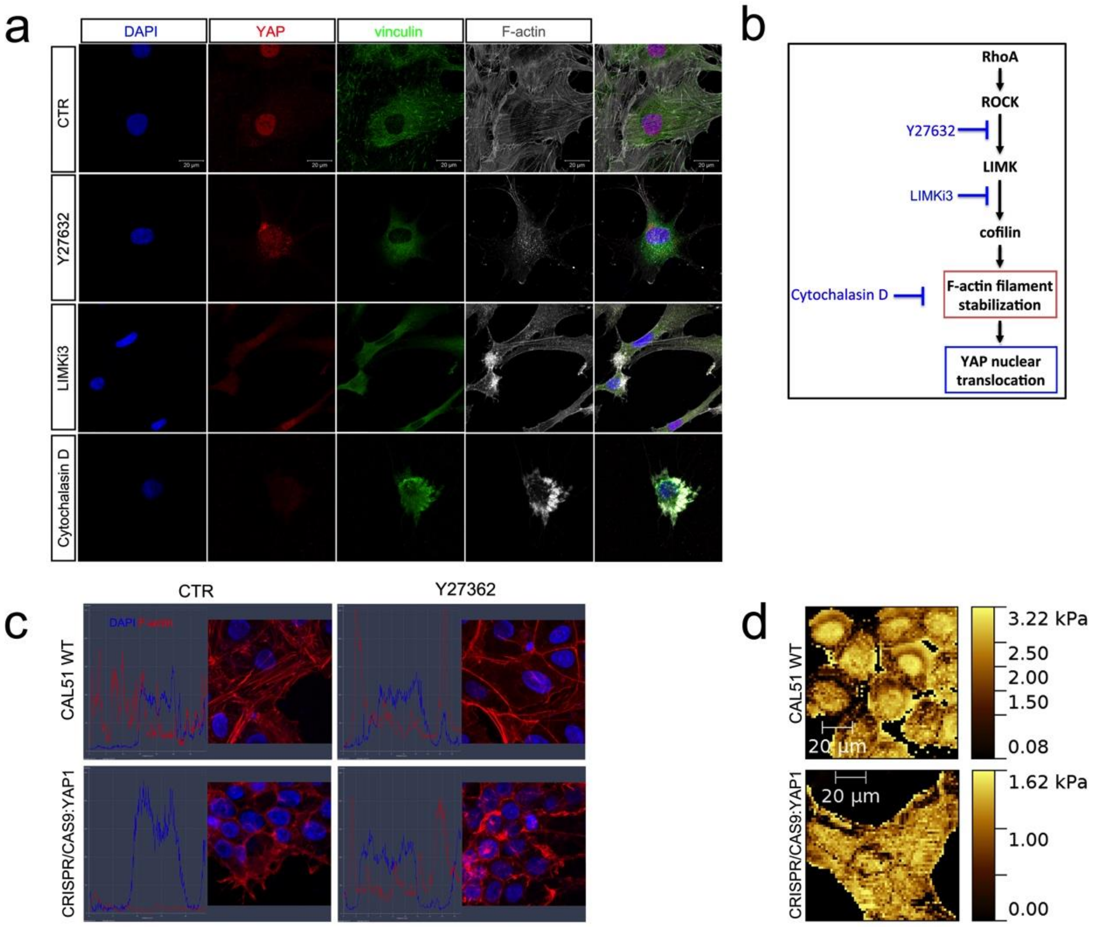

Supplementary Figure 7 | **a**, Confocal representative images of AD-MSCs treated or not with Y27632 (10 μM), LIMKi3 (20 μM), or cytochalasin D (1μM) and stained with anti-YAP (red), anti-vinculin (green) and Alexa Fluor® 647 Phalloidin (white). **e,** schematic representation of the proposed interplay between Rho/ROCK pathway and YAP nuclear shuttling, as demonstrated by selective inhibitors of Rho/ROCK pathway components. **c**, Image analysis of F-actin fiber distribution in WT CAL51, YAP-deficient clone C3 treated or not with ROCK inhibitor Y27632 (10 μM). **d**, Representative elasticity maps of wild type CAL51 and YAP mutant clone C3 as obtained by atomic force microscopy (AFM).

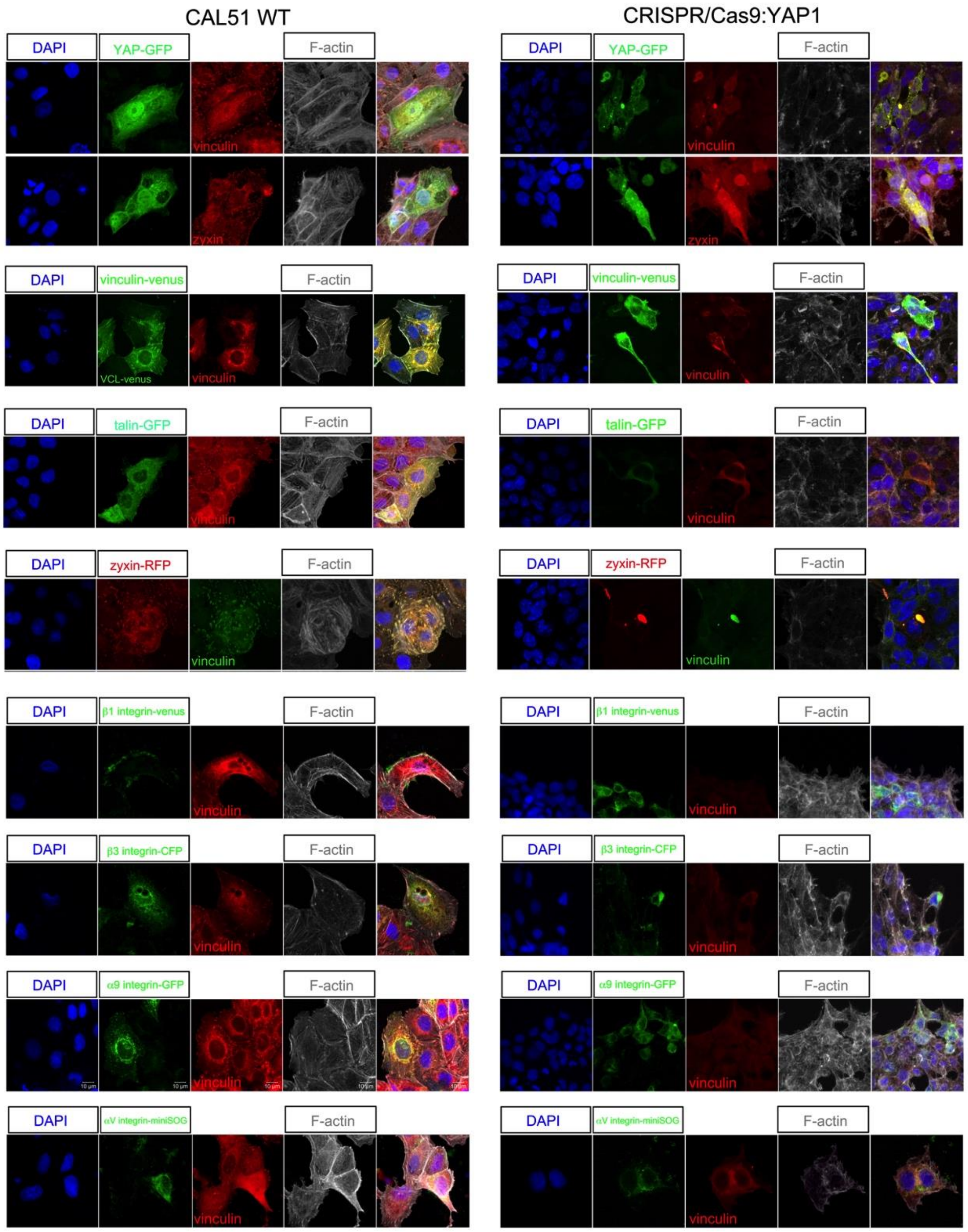

Supplementary Figure 8 | Confocal representative images of CAL51 WT and YAP mutant cells (CRISPR/CAS9:YAP1) transfected with plasmids coding for single components of Focal adhesions and stained for vinculin or zyxin. The plasmids used are the following: pEGFP-C3-hYAP1 (YAP-GFP), vinculin-venus, GFP-talin1, RFP-zyxin, mVenus-Integrin-Beta1-N-18 (β1 integrin-venus), β3-integrin-CFP, integrin alpha9 EGFP-N3 (a9 integrin-GFP), miniSOG-Alpha-V-Integrin-25 (αV integrin-miniSOG). Nuclei are counterstained with DAPI and F-actin is decorated with Alexa Fluor® 647 Phalloidin.

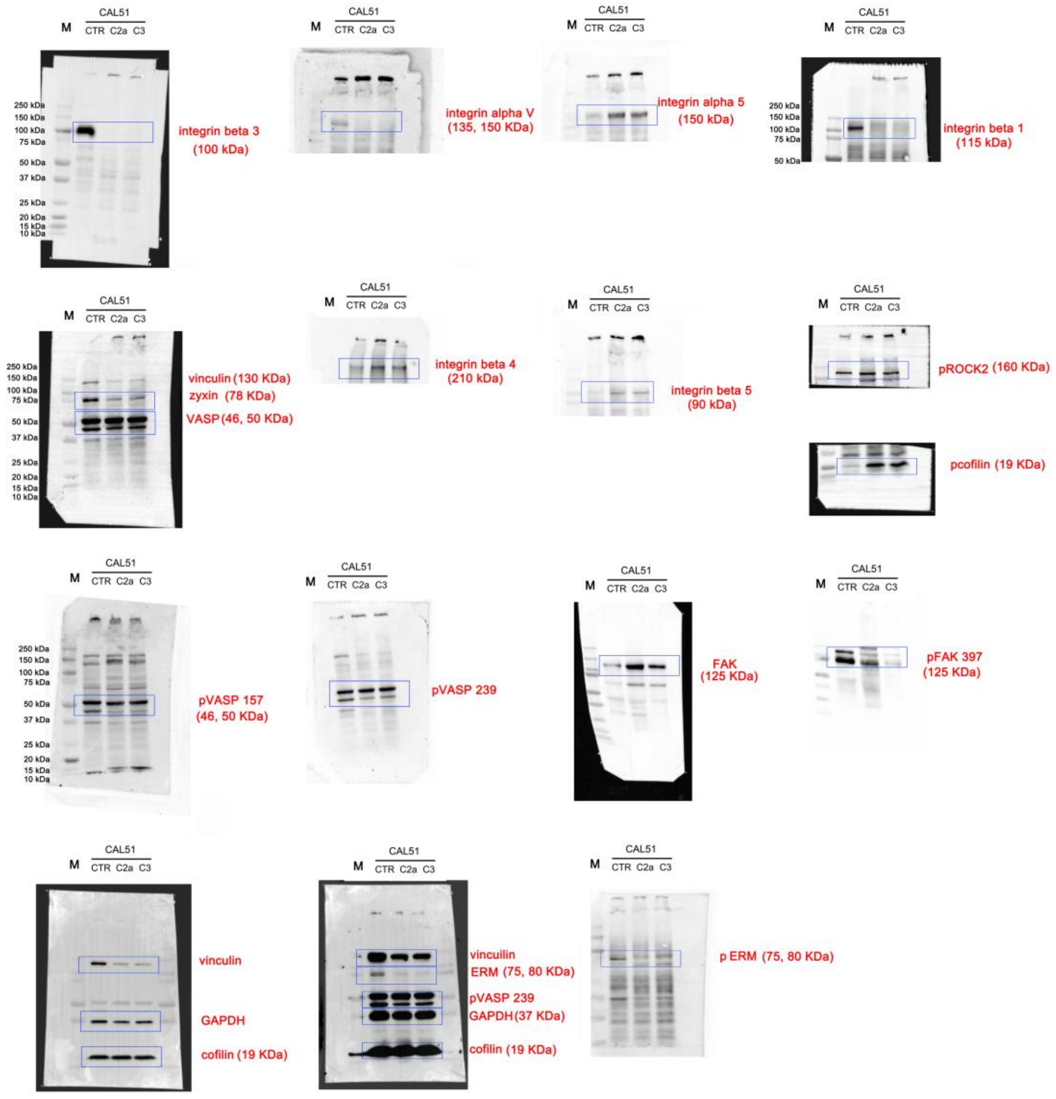

Supplementary Figure 9 | Original gel blots
